# Urinary Clusterin is a Biomarker of Renal Epithelial Senescence and Predicts Human Kidney Disease Progression

**DOI:** 10.1016/j.ekir.2025.04.035

**Published:** 2025-04-21

**Authors:** David P. Baird, Maximilian Reck, Ross Campbell, Marie-Helena Docherty, Piotr P. Janas, Tilly Mason, Zenuida Mortuza, Matthieu Vermeren, Andy Nam, Wei Yang, Nathan Schurman, Claire Williams, Jamie P. Traynor, Patrick B. Mark, Katie J. Mylonas, Jeremy Hughes, Laura Denby, Bryan R. Conway, David A. Ferenbach

**Affiliations:** 1 Centre for Inflammation Research, Institute for Regeneration and Repair, University of Edinburgh, UK; 2 Centre for Cardiovascular Science, Queen’s Medical Research Institute, University of Edinburgh, Edinburgh, UK; 3 Bruker Spatial Biology, Seattle, Washington, USA; 4 School of Medicine, Dentistry and Nursing, University of Glasgow, Glasgow, UK; 5 School of Cardiovascular and Metabolic Health, University of Glasgow, Glasgow, UK

**Keywords:** aging, biomarkers, cellular senescence, chronic kidney disease, clusterin, fibrosis

## Abstract

**Introduction:**

Cellular senescence is characterized by generally irreversible cell cycle arrest and changes in secretory activity, with senescent renal epithelia proposed as drivers of kidney fibrosis. The lack of noninvasive biomarkers represents an obstacle to the design of human trials of senescent cell–depleting medications.

**Methods:**

Proteomic analysis was performed on urine from patients with chronic kidney disease (CKD) alongside immunofluorescence staining of paired kidney biopsies (*n* = 51). Enzyme-linked immunosorbent assays (ELISAs) and immunofluorescence staining were performed in a second cohort of matched urine and kidney biopsies (*n* = 53). Spatial transcriptomic analysis was performed on kidney tissue from benign and fibrotic kidney disease (*n* = 13). Clusterin and senescence markers were analyzed *in vitro* by quantitative polymerase chain reaction (PCR) in irradiated human renal epithelia. Urinary biomarker concentrations were quantified by ELISA (*n* = 322) to assess their ability to predict patient outcomes (end-stage kidney disease or > 40% renal functional loss).

**Results:**

P21^+^Ki67^-^ epithelial senescence correlated with age and inversely with renal function. Urinary clusterin-to-creatinine ratio (uCCR) correlated tightly with P21^+^Ki67^-^ epithelial senescence in both matched urine and kidney biopsy cohorts (rho > 0.5, *P* < 0.001) and predicted levels of senescence after adjusting for other variables. Clusterin was upregulated transcriptomically in *CDKN1A* (p21) expressing epithelia *in vitro* and *in vivo*. An elevated uCCR predicted adverse renal end points in a cohort of patients with CKD after adjusting for baseline estimated glomerular filtration rate (eGFR), urinary albumin-to-creatinine ratio (uACR), age, systolic blood pressure, and sex.

**Conclusion:**

uCCR represents a surrogate for histologic quantification of p21^+^Ki67^-^ senescent renal epithelia and predicts outcomes in human kidney disease independent of existing clinical risk factors.

## Graphical abstract

**Figure:**
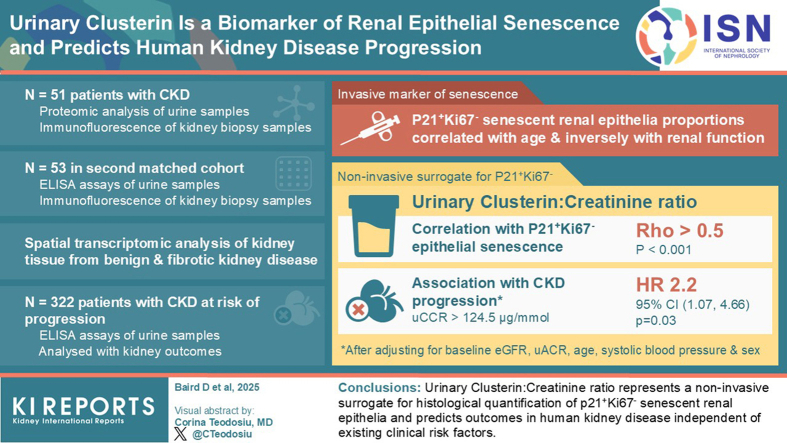

Senescent cells are transcriptionally altered, growth-arrested cells that accumulate in kidneys with advancing age and following injury. Senescent cells release profibrotic factors, collectively termed the senescence-associated secretory phenotype (SASP), that propagate injury and scarring.^1^ Accumulating evidence supports the ability of drugs that selectively deplete senescent cells (known as “senolytics”) to improve outcomes in experimental disease models,^2^ including in the kidney.^3^ With no noninvasive biomarkers available, quantifying senescence in human solid organs requires a tissue biopsy. This represents a major obstacle to designing human trials of therapies that target senescent cells.

Levels of *CDKN1A* transcript (encoding the cyclin-dependent kinase inhibitor P21, known to inhibit G1/S progression) in the kidney correlate with worsened renal function in human CKD,^3^ with elevated P21 (in the absence of proliferation markers such as Ki67) used to identify senescent epithelia in clinical research settings.^4^ The Kidney Precision Medicine Project dataset demonstrated upregulated *CDKN1A* (P21) expression in “late-adaptive” proximal tubular epithelia in human CKD.^5^ Our multiomic atlas of human kidney disease confirmed that *CDKN1A* (P21) expression was upregulated in a proximal tubular subset, expressing key differentially expressed genes of the Kidney Precision Medicine Project “late-adaptive” proximal tubular cells alongside multiple senescence-associated differentially expressed genes.^6^

Here, we addressed the hypothesis that P21**^+^**KI67**^-^** senescent renal epithelia could be quantified noninvasively by detecting their selectively secreted proteins in human urine samples and then determined if these noninvasive surrogates of senescence predicted outcomes in patients with CKD.

## Methods

### seNSOR Biobank Recruitment

**Figure 1:**
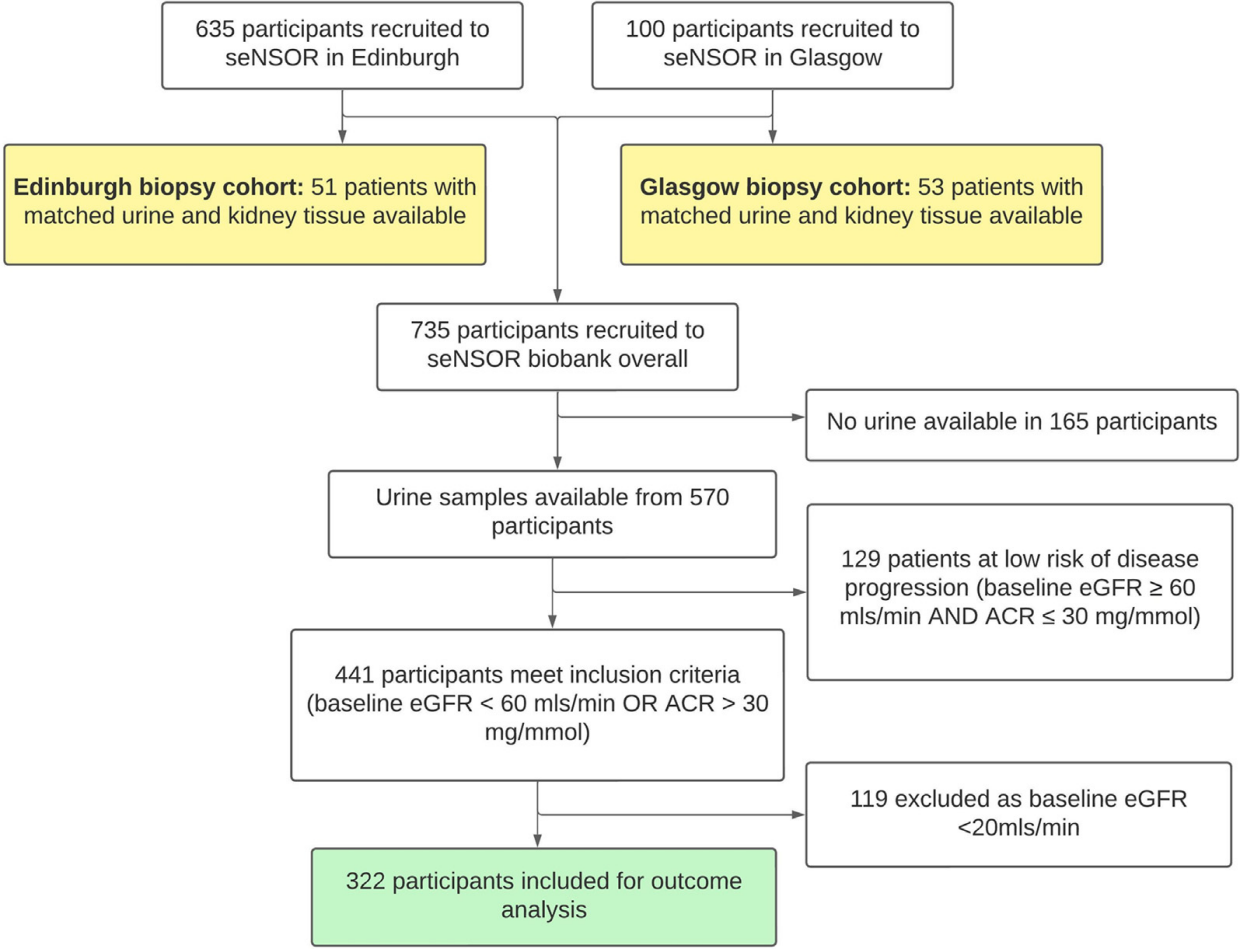
Overview of participants in the seNSOR biobank and composition of the Edinburgh biopsy cohort, Glasgow biopsy cohort, and outcome analysis cohort, respectively.

The seNSOR biobank (full study title: “non-invasive biomarkers of renal disease”) comprised 635 patients recruited from renal clinics at the Royal Infirmary of Edinburgh, Edinburgh between March 2017 and March 2019. In addition, 100 patients with CKD were recruited to seNSOR from the Queen Elizabeth University Hospital, Glasgow between March 2018 and August 2019 (Figure 1). Ethical approval was obtained from the Offices for Research Ethics Committee (REC/15/ES/0094, REC/20/ES/0061, REC 14/WS/1035, and REC 22/WS/0020) and informed patient consent and anonymization undertaken in line with the Uniform Requirements of the International Committee of Medical Journal Editors as described previously. Participant information was collected upon enrolment with urine samples snap-frozen and stored at −80 °C.

Participant information included age, ethnicity, blood pressure, and etiology of CKD. Glomerular filtration rate was estimated from baseline and follow-up serum creatinine using the 2009 CKD Epidemiology Collaboration equation.^7^

### Patient Selection for Biopsy Cohorts

The Edinburgh biopsy cohort of 51 participants included all those recruited in Edinburgh with kidney biopsy tissue and urine samples from the same time point available for analysis. The Glasgow biopsy cohort included 53 participants who were recruited in Glasgow and had matched kidney biopsy tissue and urine samples available. All kidney biopsies were performed for clinical indications by physicians independent of the research team. Clinical parameters including age, baseline eGFR, and ACR were obtained at the time of biopsy.

### Patient Selection for Outcome Analysis

Urine samples were available from 570 participants recruited into the seNSOR biobank. One hundred twenty-nine patients at low risk of CKD progression (baseline eGFR ≥ 60 ml/min and ACR ≤ 30 mg/mmol) were excluded. This matches the criterion used by other CKD biobanks such as the National Unified Renal Translational Research Enterprise (NURTuRE) biobank.^8^ An additional 119 with baseline eGFR < 20 ml/min were excluded because they had already reached or were close to the study end point. Three hundred twenty-two participants were included in the outcome analysis.

### Human Kidney Tissue From Outwith seNSOR Biobank

Four nephrectomy specimens from patients with fibrosis due to recurrent pyelonephritis were included in the spatial transcriptomic analysis. In addition, for the clusterin/P21 immunofluorescence staining, *n* = 5 nephrectomy samples were used from patients with transitional cell carcinoma of the ureter leading to ureteric obstruction as determined by the presence of hydronephrosis on computed tomography imaging. Use of tissue was approved by the steering committee of the National Research Scotland Lothian Bioresource (REC 20/ES/0061, studies SR1651, SR1887).

### Sex as a Biological Variable

Our study examined male and female human patients with CKD. Sex was as reported by participants at time of recruitment.

### Liquid Chromatography With Tandem Mass Spectrometry Analysis

Liquid Chromatography With Tandem Mass Spectrometry **(**LC-MS/MS) studies were undertaken on all urine samples in the Edinburgh biopsy cohort by Lisa Imrie and Tessa Moses from the Edinomics Team (University of Edinburgh). Samples were depleted of high abundance proteins (serum albumin and IgG) using Agilent multiple affinity removal spin cartridges following the manufacturer’s protocol. They were trypsin-digested using S-TrapTM (Protifi) following the manufacturer’s protocol. After speed vac drying, peptide samples were resuspended in MS-loading buffer (0.05% v/v trifluoroacetic acid in water) and 50 pmol of MassPREP alcohol dehydrogenase digestion standard (Waters) was spiked into each sample (added as an external standard). They were then filtered using Millex filter before high-performance LC-MS/MS analysis.

Nano-electrospray ionization–high-performance LC-MS/MS analysis was performed using an online system of a nano- high-performance LC (Dionex Ultimate 3000 RSLC, Thermo-Fisher Scientific) coupled to a QExactive mass spectrometer (Thermo-Fisher Scientific) with a 300 μm × 5 mm precolumn (Acclaim Pepmap, 5 μm particle size) joined with a 75 μm × 50 cm column (EASY- Spray, 3 μm particle size). The nano-pump was run using solvent A (2% acetonitrile in water 0.1% formic acid) and solvent B (80% acetonitrile-20% water and 0.1% formic acid) and peptides were separated using a multistep gradient of 2%–98% buffer B at a flow rate of 300 nl/min over 90 minutes. Progenesis (version 4 Nonlinear Dynamics, UK) was used for LC-MS/MS label-free quantitation. Filtering was carried out so that only MS/MS peaks with a charge of 2+, 3+, or 4+ were taken into account for the total number of “features” (signal at 1 particular retention time and m/z) and only the 5 most intense spectra per “feature” were included. MS/MS spectra were searched using MASCOT version 2.4 (Matrix Science Ltd.) against a UniProt *H.sapiens* database with maximum missed-cut value set to 2. The following parameters were used in all searches: (i) variable methionine oxidation, (ii) fixed cysteine carbamidomethylation, (iii) precursor mass tolerance of 10 ppm, (iv) MS/MS tolerance of 0.05 Da, (v) significance threshold (*P*) < 0.05, and (vi) final peptide score of 20. Only proteins with 2 or more unique peptides were considered.

### ELISA Analysis

Urine clusterin was measured on stored urine samples using R&D Duoset ELISAs (R&D Systems, Minneapolis, MN, catalogue number DY5874) with all samples run in duplicate. Based on pilot studies, most samples were analyzed following 1:1000 predilution, with any results outside the published working range of the assay rerun at 1:100, 1 in 10,000, or 1:100,000 dilution.

### Biochemical Assays

Urinary creatinine measurements were determined using the creatinase enzymatic method making use of a commercial kit (17654H, Sentinel Diagnostics via Alpha Laboratories Ltd., Eastleigh, UK) adapted for use on either a Cobas Fara or Mira analyser (Roche). Intraassay precision was < 3% whereas interassay precision was coefficient of variation <5%. Urinary clusterin was corrected for urinary creatinine throughout.

Microalbumin measurements were determined using a commercial kit (#1 0242 99 10 021, DiaSys Diagnostic Systems) adapted for use on a Cobas Mira analyser (Roche). This immunoturbidimetric assay was standardized against purified mouse albumin standards (Sigma Aldrich) with samples diluted in deionized water as appropriate. Intraassay precision was < 5% whereas interassay precision was < 7.1%.

### Tissue Biopsy Staining

Immunofluorescence staining on human kidney biopsy samples, which included cortex and outer medulla, was performed using a BOND III automated immunostainer (Leica Biosystems) using sequential tyramide-coupled fluorophores. Antibody concentrations were determined by titration of single immunofluorescent stains to determine the most suitable dilution before being combined into a multiplex stain. The P21/KI67 immunofluorescent stain comprised 4 antibodies. Pancytokeratin (CKPAN, Merck, Cat C2562) at 1:6000 dilution, and CD10 (Leica, Cat: NCL-L-CD10-270) at 1:600, both tubular epithelial markers, were added together, and the same Opal 520 fluorophore used for both. Other antibodies used in sequence targeted KI67 (Agilent, Cat M724001-2) with an Opal 650 fluorophore and P21(Abcam, Cat ab109520) with an Opal 570 fluorophore (all secondary antibodies used at 1:500 dilution). For the P21/clusterin staining, clusterin (Abcam, Cat ab92548) was substituted into the panel at 1:500 dilution in place of KI67 (using the Opal 650 fluorophore) with P21, pancytokeratin, and CD10 included as described above.

Cortical slides stained for Masson’s Trichrome were available from 49 out of 51 participants in the Edinburgh biopsy cohort and 50 out of 53 patients in the Glasgow biopsy cohort. A single blinded operator (BC) performed semiquantitative cortical fibrosis assessment on these slides.

### Human Renal Proximal Tubular Epithelial Cells (hRPTEC) Cell Culture

hRPTEC (American Type Culture Collection) were maintained in Dulbecco’s modified Eagle’s medium/F-12 with GlutaMAX (Thermo Fisher, Cat 31331028) and supplemented with a human telomerase reverse transcriptase immortalized RPTEC growth kit (American Type Culture Collection, ACS-4007), Pen-Strep, and Geneticin (50 mg/ml) (Thermo Fisher, Cat 10131035). Cells were grown at 37 °C in 5% CO_2_. hRPTECs were plated in 12-well culture plates at 2.4 × 10^4^ cells (control group) or 4.8 × 10^4^ cells (irradiated group) per well. After 72 hours of culture, hRPTECs were exposed to 10-Gy radiation to induce senescence. Control hRPTECs were taken to the irradiator but not placed inside. Culture medium was changed immediately following irradiation, then every 2 to 3 days. Seven days after irradiation, RNA was extracted with TRIzol.

### hRPTEC RNA Extraction and Quantitative PCR

RNA was extracted from hRPTECs using TRIzol (Thermo Fisher, Cat 15596026) and reverse transcribed to cDNA with QuantiTect Reverse Transcription Kit (Qiagen, Cat 205314) according to the manufacturer’s instructions. Quantitative PCR was carried out using PerfeCTa FastMix II (Quantabio, Cat 95118-012) and TaqMan Gene Expression Assays (Thermo Fisher, Cat 4331182) on a QuantStudio 5 real-time PCR instrument. TaqMan assays used were: *CDKN1A* (Hs00355782_m1), *CLU* (Hs00156548_m1), *HPRT1* (Hs02800695_m1), *MKI67* (Hs04260396_g1), and *PPIA* (Hs04194521_s1). mRNA expression was normalized for *HPRT1* and *PPIA* (average) expression and presented as relative expression (2^ΔΔCt^) to nonirradiated, proliferating controls.

### Microscopy and Image Analysis

For the P21/KI67 staining, images of whole slides were acquired using the Axio Scan Z1 whole slide scanner (Zeiss, Jena, Germany). Images were then analyzed using QUPath (version 0.3.2). Cells were classified automatically using the following algorithm based on nuclear staining of P21 and KI67 as follows: P21 positive/KI67 negative, P21 negative/KI67 positive, double positive or double negative. Only tubular epithelia that were P21 positive and KI67 negative were classed as having a cell cycle inhibitor profile consistent with senescence and this number was expressed as a percentage of the total number of tubular cells. In participants where > 1 section from their biopsy was available, the cell counts from each section were combined before percentages were calculated.

For the P21/clusterin staining, images were acquired using the Zeiss Observer scanner (Zeiss, Jena, Germany). Images were then analyzed using QUPath (version 0.5.1). Cells were classified based on nuclear staining of P21 and cytoplasmic staining for clusterin.

### Spatial Transcriptomic Analysis

Spatial transcriptomic analysis was performed on kidney tissue from 13 patients; this included 9 core biopsies from patients in the Edinburgh biopsy cohort (*n* = 3 with minimal change disease and *n* = 6 with IgA nephropathy) and an additional 4 nephrectomy specimens from patients with fibrosis due to recurrent pyelonephritis. CDKN1A and CLU expression in subcellular resolution spatial transcriptomics data (Bruker Spatial Biology CosMx SMI) was analyzed using preprocessed data downloaded in Seurat (4.4.0) format from gene expression omnibus (GSE253439). The original cell annotations were used to classify proximal tubule, or loop of Henle and distal convoluted tubule cells which express CDKN1A (independent of cell state).

To avoid false positive classifications due to noise in the assay and cell segmentation errors, cells were considered CDKN1A+ when ≥ 2 CDKN1A transcripts were detected within cell segmentation boundaries.

Spatial enrichment of CLU transcripts in relation to cell centroids of a given cell type were calculated as described previously.^5^ Briefly, for each individual cell, a search radius of 50 mm in 1 mm steps from the cell centroid was defined. For each given cell type, the number of detected transcripts (of a given gene) were summed and normalized by the area of the circle segment and the number of cells encountered in the search area. Simultaneously, to calculate the background signal in the general cell population, 10,000 random cells were selected and the normalized transcript counts were calculated as before. The enrichment ratio at each interval was then defined as the log2+1-fold enrichment ratio of the query cell type over the randomly selected cell population. Cell boundaries and transcripts in 2-dimensional coordinates were visualized using the Seurat ImageDimPlot function.

Differential gene expression between CDKN1A+ and CDKN1A− proximal tubular cells was assessed using the Wilcoxon signed-rank test implemented by the Seurat function FindMarkers() with default parameters.

### Statistical Tests

Normality was assessed using the Shapiro-Wilk test for all variables. Clinical characteristics for continuous data were expressed as mean ± SD when data was normally distributed and median (interquartile range) when not normally distributed. Categorical variables were expressed as counts. When comparing 2 unpaired groups, a *t* test was used when the data was normally distributed, and a Mann-Whitney test used if the data was not normally distributed. Categorical values were assessed using a chi-square (χ2)-test. For contingency tables, Fisher exact test was used.

For the LC-MS/MS data, values were corrected for alcohol dehydrogenase and then for urinary creatinine. To determine the linear correlation between each protein detected and histologic P21^+^KI67^-^ epithelial cell proportions, correlation coefficients (rho) were estimated using Spearman’s rank tests because the data was not normally distributed. Adjusted *P* values were calculated using the Benjamini and Hochberg False Discovery Rate method from the list of *P* values, generated from the correlation between P21^+^KI67^-^ epithelial cell proportions and protein levels.

For quantitative PCR analysis of *in vitro* cell culture data, relative expressions of *CDKN1A*, *MKI67*, and *CLU* in irradiated hRPTECs were compared with proliferating controls normalized for HPRT1 and PPIA (average) expression, and Welch's *t* test performed.

Linear regression was used to determine if levels of log2-tranformed clusterin predicted histologic P21^+^KI67^-^ epithelial cell proportions as the dependent variable in models alongside baseline eGFR, ACR, and patient age. No adjustment for multicollinearity was required (variance inflation factors were ≤ 2.1 for variables in each model).

Receiver operating characteristic curve analysis, including data from the Edinburgh and Glasgow biopsy cohorts was used to determine the optimal uCCR levels cutoff point to discriminate between those with top tertile of P21^+^KI67^-^ senescent renal epithelial senescence levels and the remaining participants.

For the outcome analysis, CKD progression was defined as reaching end-stage kidney disease (starting renal replacement therapy or maintaining an eGFR < 15 ml/min for > 90 days) or > 40% reduction in renal function from eGFR at baseline (maintained for > 90 days).^9^^,^^10^ Kaplan-Meier survival curves were constructed with the log-rank test used to compare curves. Univariate and multivariate analyses of outcomes using Cox proportional hazards survival models were performed. Death was treated as a censoring event. The proportional hazards assumption was tested and valid. No adjustment for multicollinearity was required. A *P* value < 0.05 was considered significant.

All analyses were performed using R version 4.1.2 (R Foundation for Statistical Computing, Vienna, Austria) and GraphPad Prism versions 9 and 10 (GraphPad Software, Boston, MA, USA).

## Results

### Senescence in Human Kidney Tissue

**Figure 2:**
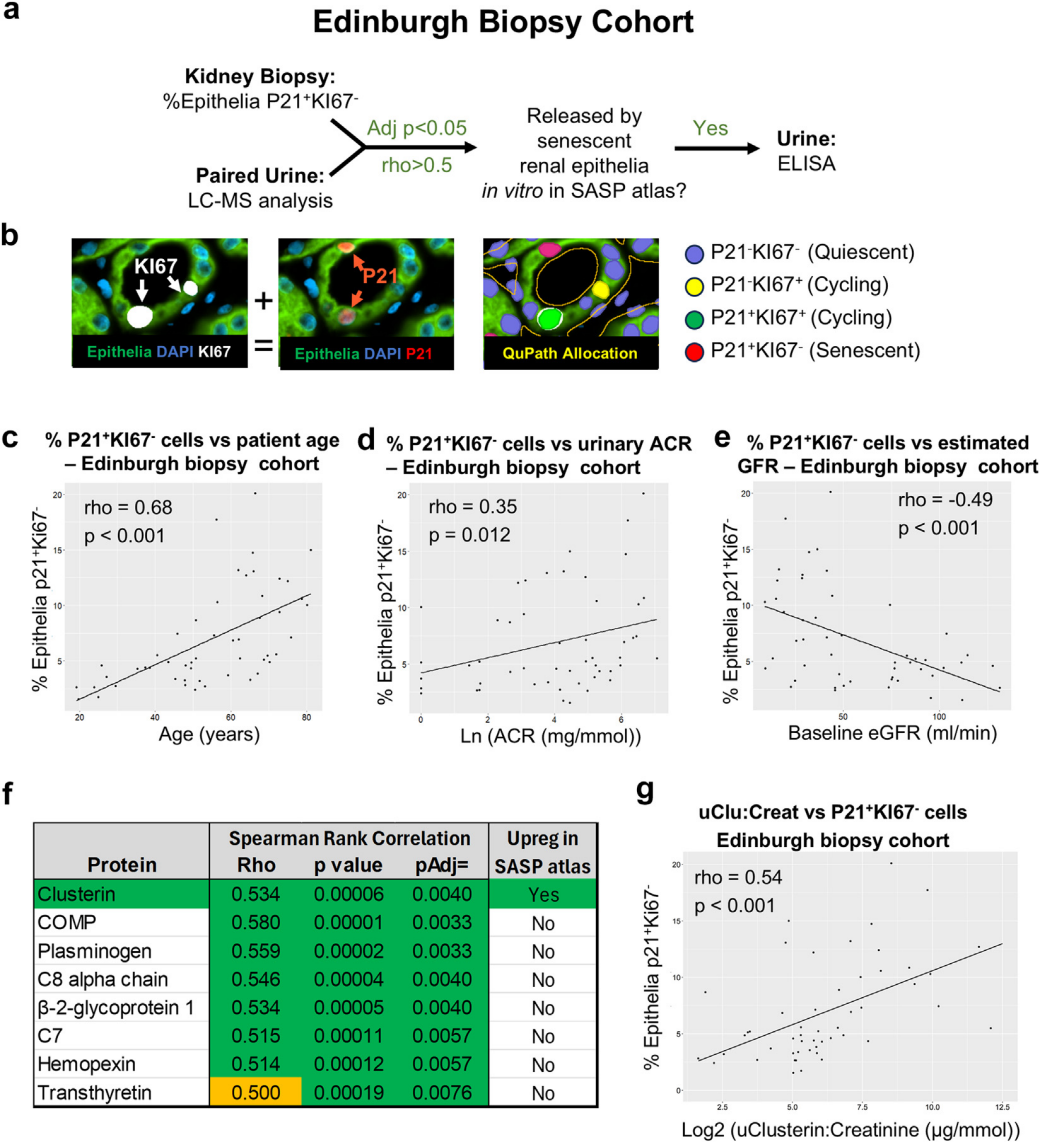
(a) Experimental schema for immunofluorescent staining of human biopsies and LC-MS/MS and ELISA analysis of paired urine samples from the Edinburgh biopsy cohort (*n* = 51). (b) Immunofluorescent staining and automated analysis of human renal biopsies to quantify senescent (P21^+^KI67^-^) cells. Correlations in the Edinburgh cohort between the proportion of P21^+^KI67^-^ senescent epithelial cells, and (c) age, (d) Ln(ACR) and (E) eGFR. (f) Urinary proteins in LC-MS/MS analysis correlating most closely with the proportion of P21^+^KI67^-^ senescent epithelial cells. SASP Atlas status as defined by Basisty *et al.*^11^ (g) Correlation between the proportion of P21^+^KI67^-^ senescent epithelial cells and urinary clusterin-to-creatinine in the Edinburgh biopsy cohort. ACR, albumin-to-creatinine ratio; eGFR, estimated glomerular filtration rate; ELISA, enzyme-linked immunosorbent assay; LC-MS/MS, liquid chromatography with tandem mass spectrometry; SASP, senescence associated secretory phenotype.

We analyzed 51 kidney biopsies from patients with paired urine samples in the seNSOR CKD biobank recruited in Edinburgh (Edinburgh biopsy cohort; Figure 2a, Table 1). Immunofluorescence staining was performed for P21, KI67, and CD10+pancytokeratin as senescence, proliferation, and pan-epithelial cell markers, respectively (Figure 2b). There was a wide range in age and baseline eGFR among participants (median eGFR at recruitment was 45.6 [range: 9.2–131.2] ml/min per 1.73 m^2^,S1 median age: 56.2 [range 19–81] years). The median percentage of P21**^+^**KI67**^-^** senescent epithelia was 5.2% (interquartile range: 3.6%–9.7%). Senescent epithelial proportions correlated with increasing patient age and uACR, and inversely with eGFR (Figure 2c–e).

**Table 1:** Baseline characteristics of the Edinburgh biopsy cohort and Glasgow biopsy cohort

Characteristics	Edinburgh biopsy cohort	Glasgow biopsy cohort	*P-*value for comparison
*n*	51	53	
Age (yrs), median (IQR)	56.2 (45.6–67.4)	54.9 (37.1–65.3)	0.61
Male/Female, *n*	32/19	29/24	0.53
eGFR, (ml/min per 1.73 m^2^), median (IQR)	45.6 (28.8–83.8)	47.1 (32.6–84.2)	0.61
SBP, mean ± SD	139 ± 21.8	139.5 ± 20.5	0.9
DBP, mean ± SD	80.2 ± 12.4	82.5 ± 13.6	0.37
uACR (mg/mmol), median (IQR)	85.9 (16.4–247.7)	131.2 (29.2–333.2)	0.14
Ethnicity,			
White, *n*	47	43	
Black, *n*	1	0	
Asian, *n*	1	3	
Not recorded, *n*	2	7	
Diagnoses			
IgA nephropathy	21	9	
Membranous nephropathy	0	7	
Interstitial nephritis	6	8	
Minimal change disease	5	1	
Primary FSGS	0	4	
Diabetic nephropathy	5	3	
Vasculitis	5	3	
Hypertensive / ischemic nephropathy	2	5	
Lupus nephritis	2	3	
Other^a^	5	10	

CKD, chronic kidney disease; DBP, diastolic blood pressure; eGFR, estimated glomerular filtration rate; FSGS, focal segmental glomerulosclerosis; IQR, interquartile range; SBP, systolic blood pressure; uACR, urinary albumin-to-creatinine ratio.

a Other diagnoses in discovery cohort: Henoch-Schönlein purpura (*n* = 2), lithium toxicity, obesity glomerulopathy and myeloma; and validation cohort: mesangiocapilllary glomerulonephritis (*n* = 2), thin glomerular basement membrane (*n* = 2), Henoch-Schönlein purpura, AA amyloid, AL amyloid, infiltration by lymphoma, CKD of uncertain etiology, and acute kidney injury.

### Senescence Biomarker Identification

To identify urinary proteins correlating with tubular cell senescence, high-performance LC-MS/MS analysis was performed on all 51 paired urine samples. Three hundred thirty-one distinct proteins were detected by LC-MS/MS (Supplementary Table S1), of which 81 had a positive correlation with the proportion of P21**^+^**KI67**^-^** senescent epithelia (adjusted *P* < 0.05) and 8 having a correlation coefficient (rho) > 0.5 (Figure 2f). Results were compared with proteins upregulated in the ”SASP atlas” of senescent human renal epithelia *in vitro*.^11^ Urinary CLU protein (uClusterin) correlated tightly, with the proportion of P21**^+^**KI67**^-^** epithelial senescence *in vivo* (rho > 0.53, *P* < 0.001) and was the only protein with correlation > 0.5 that was upregulated in the SASP atlas of renal epithelial senescence *in vitro*.^11^ uClusterin was quantified using ELISA in this Edinburgh biopsy cohort and corrected for urinary concentration using urinary creatinine (Figure 2g). Multivariate analysis demonstrated that uCCR predicted P21^+^KI67^-^ senescent renal epithelia levels independently of eGFR, age and uACR (Table 2).

**Table 2:** Multiple linear regression analysis of the utility of baseline parameters to predict P21^+^KI67^-^ senescent renal epithelial cell proportions in biopsies from patients with CKD recruited to the Edinburgh cohort

Edinburgh biopsy cohort	Estimate	*P* value
Log_2_ (clusterin-to-creatinine ratio)	0.60	0.039
Baseline eGFR (ml/min)	−0.03	0.117
Age (yrs)	0.10	0.004
ACR (mg/mmol)	0.0001	0.798

ACR, albumin-to-creatinine ratio; CKD, chronic kidney disease; eGFR, estimated glomerular filtration rate.

**Figure 3:**
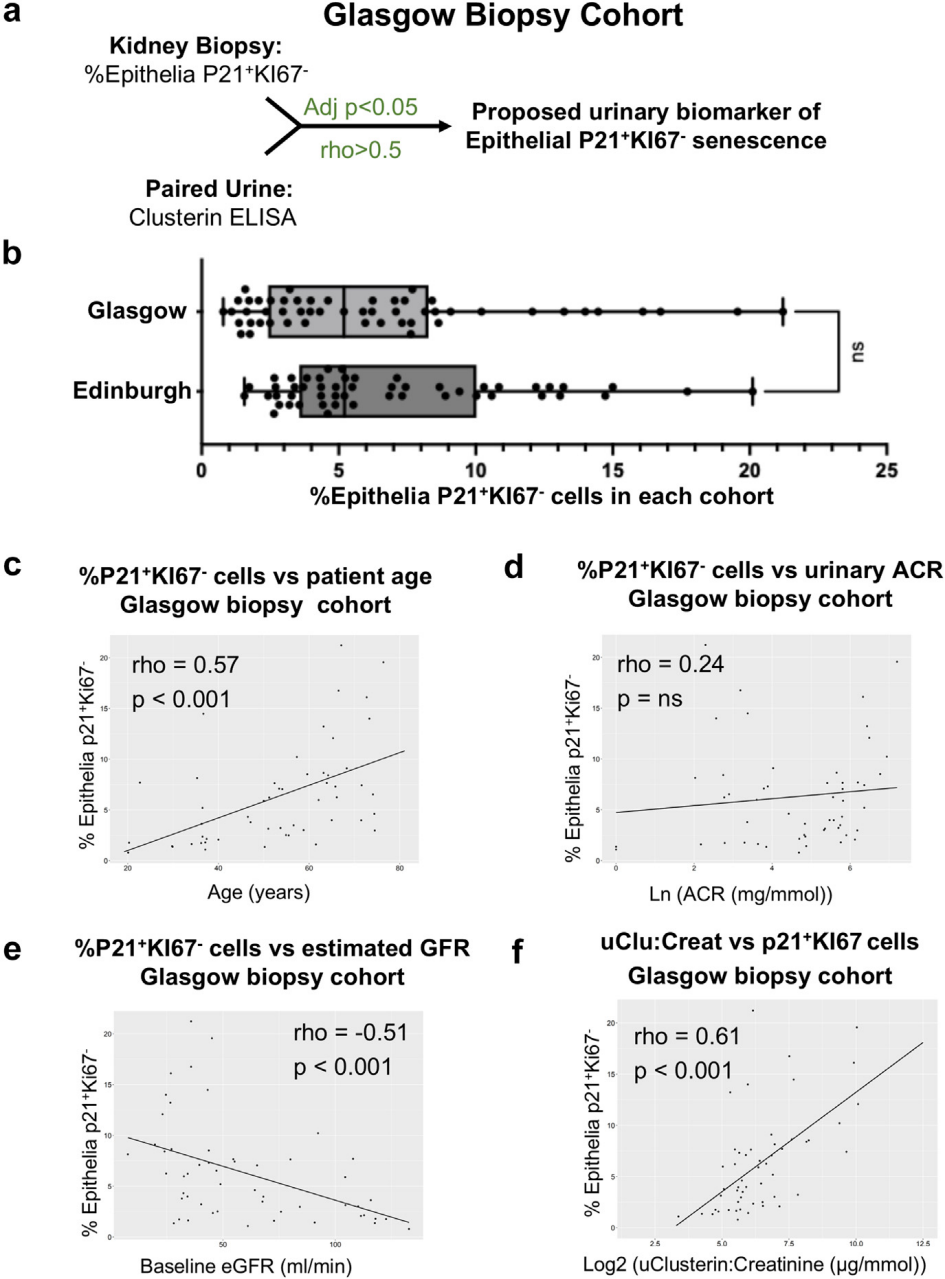
(a) Experimental schema for immunofluorescent staining of human biopsies and ELISA analysis of paired urine samples from the Glasgow biopsy cohort (*n* = 53). (b) Comparison of P21^+^KI67^-^ senescent epithelial cells between the Edinburgh and Glasgow biopsy cohorts. Correlations in the Glasgow cohort between the proportion of P21^+^KI67^-^ senescent epithelial cells and (c) age, (d) Ln(ACR) and (e) eGFR. (f) Correlation between the proportion of P21^+^KI67^-^ senescent epithelial cells and urinary clusterin-to-creatinine in the Glasgow biopsy cohort; ACR, albumin-to-creatinine ratio; eGFR, estimated glomerular filtration rate; ELISA, enzyme-linked immunosorbent assay.

We confirmed our findings in a cohort of patients with CKD recruited to the seNSOR biobank in Glasgow (the Glasgow biopsy cohort) (Figure 3a). This cohort had similar proportions of P21**^+^**KI67**^-^** senescent epithelia (median: 5.2%, interquartile range: 2.5%–8.1%) and baseline characteristics to those in the Edinburgh biopsy cohort (Figure 3b, Table 1). P21**^+^**KI67**^-^** senescent epithelia proportions again correlated with age and inversely with renal function; though in this cohort, the relationship with uACR was not statistically significant (Figure 3c–e). uCCR again correlated strongly with P21**^+^**KI67**^-^** senescent epithelial cell proportions (rho = 0.61, *P* < 0.001; Figure 3f), with adjusted uCCR improving prediction of histologic senescence independently of eGFR, uACR, and age (Table 3).

**Table 3:** Multiple linear regression analysis of the utility of baseline parameters to predict P21^+^KI67^-^ senescent renal epithelial cell proportions in biopsies from patients with CKD recruited to the Glasgow cohort

Glasgow biopsy cohort	Estimate	*P* value
Log_2_ (clusterin-to-creatinine ratio)	1.47	0.005
Baseline eGFR (ml/min)	−0.04	0.05
Age (yrs)	0.07	0.08
ACR (mg/mmol)	0.0001	0.96

ACR, albumin-to-creatinine ratio; CKD, chronic kidney disease; eGFR, estimated glomerular filtration rate.

In both the Edinburgh and Glasgow biopsy cohorts, uCCR levels correlated with the degree of cortical fibrosis (Edinburgh biopsy cohort: rho = 0.38, *P* = 0.008; Glasgow biopsy cohort: rho = 0.33, *P* = 0.017; Supplementary Figure S1).

### Clusterin Expression

**Figure 4:**
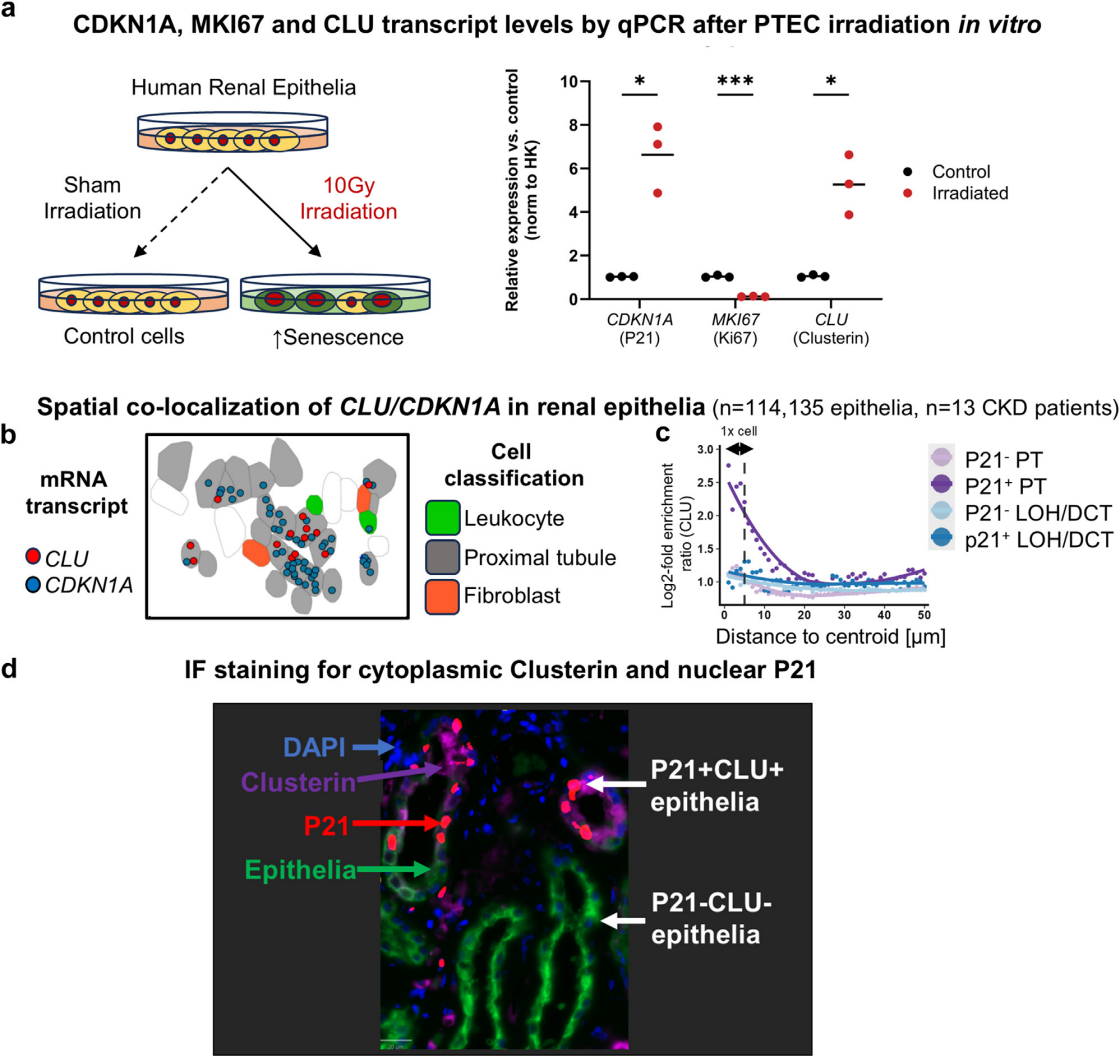
(a) Relative expression of *CDKN1A*, *MKI67*, and *CLU* in irradiated hRPTECs (*n* = 3) compared with proliferating controls (*n* = 3). Normalized for HPRT1 and PPIA (average) expression. Individual data points plotted with mean. Welch's *t* test performed (∗*P* < 0.05, ∗∗∗*P* < 0.0005). (b) Image of CLU/CDKN1A colocalization by spatial transcriptomic analysis. (c) Spatial enrichment (log2 scale) of CLU transcripts in proximity to P21^+^ and P21^-^ renal proximal tubule (PT) or loop of Henle/distal convoluted tubule (LOH/DCT) epithelial cells. (d) Immunofluorescent staining for P21 and clusterin in human kidney biopsies with channels visible as per image label. hRPTEC, human renal proximal tubular epithelial cells.

Studies have shown increased CLU protein secretion from human cells undergoing senescence *in vivo* in other organs.^12^ To seek further evidence of CLU expression in senescent renal epithelial *in vitro*, cultures were performed using primary hRPTECs. Irradiation-induced senescent epithelia demonstrated decreased proliferation, with reduced *MKI67* (*P* < 0.0005 vs. proliferating controls) and increased *CDKN1A* and *CLU* (*P* < 0.05 vs. proliferating controls; Figure 4a). To determine whether *CLU* production was selectively increased in CDKN1A+ senescent renal epithelia, subcellular resolution spatial transcriptomic analysis (CosMx platform) was performed on kidney tissue from 13 patients with CKD. *CLU* transcript levels were enriched within *CDKN1A*-expressing proximal tubular epithelia on automated counting and colocalization analysis (*n* = 114,135 epithelia, adjusted *P* = 3.91 x 10^−8^; Figure 4b and c). This pattern of CDKN1A+/CLU+ coenrichment was also present at a protein level on multiplex immunofluorescent staining and analysis of *n* = 5 obstructed human kidneys (*n* = 230,028 epithelial cells; Figure 4d). The odds ratio for P21+ cells expressing clusterin was 2.14 compared with P21 negative cells (95% CI: 2.04–2.24, *P* < 0.0001 by Fisher exact test, Supplementary Table S2) though clusterin expression was not limited to p21+ cells.

### Clusterin Predicts Poor Outcomes in CKD

**Figure 5:**
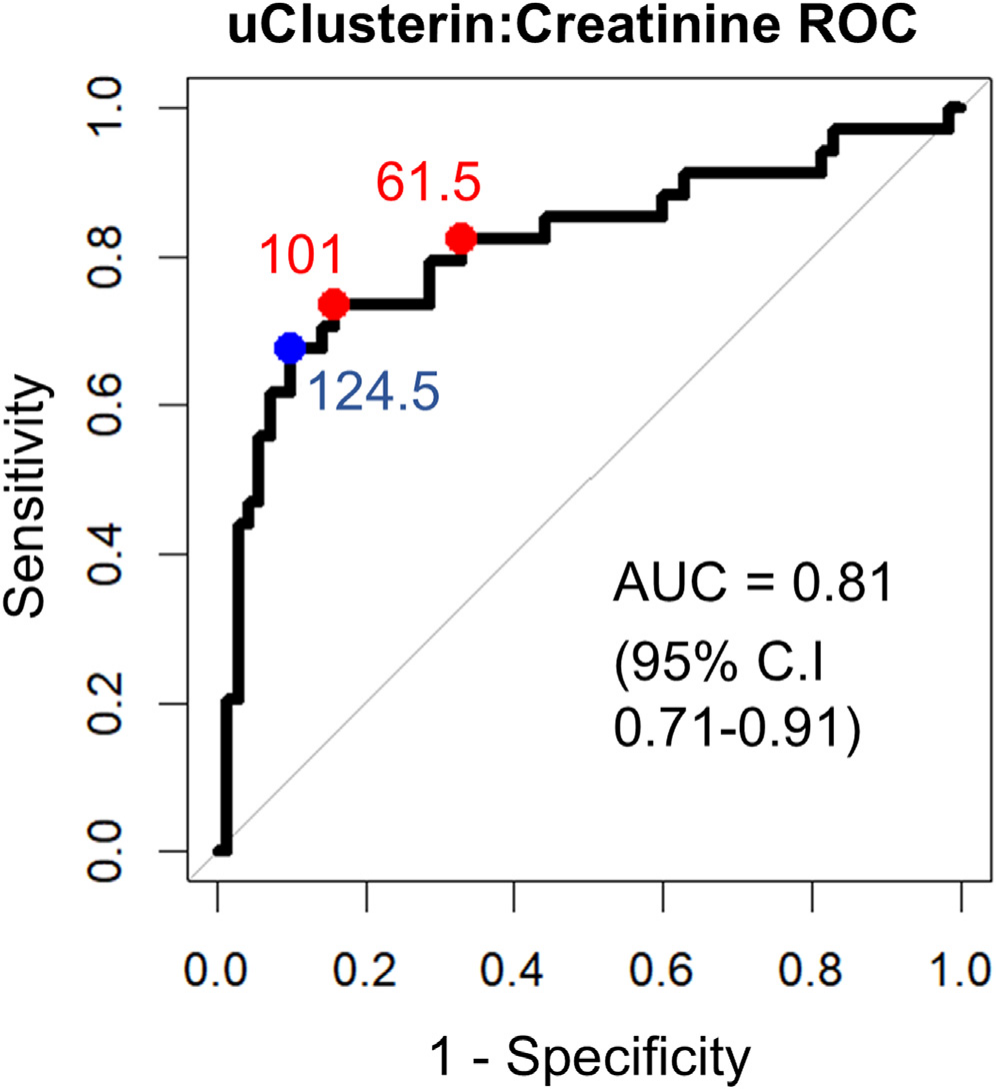
Receiver operating characteristic curve for urinary clusterin-to-creatinine results discriminating between those in the highest tertile of P21^+^KI67^-^ senescent epithelial proportions and other participants. Selected thresholds (as indicated by labels) illustrated on curve AUC = 0.81 (95% CI: 0.71–0.91, *P* < 0.001 compared with null AUC of 0.5). AUC, area under the curve.

Next, we explored the optimal uCCR required to identify patients with high levels of P21**^+^**KI67**^-^** senescent epithelia. On the receiver operating characteristic, the area under the curve was 0.81 (95% CI: 0.71–0.91, *P* < 0.001 compared with null area under the curve of 0.5) (Figure 5). By prioritizing high specificity over high sensitivity, a threshold of 124.5 μg/mmol uCCR was selected. This corresponded to a sensitivity of 68% and specificity of 90% for identifying patients in the highest tertile of P21**^+^**KI67**^-^** senescent epithelia proportions. Alternative threshold values were also considered; uCCR levels > 101 μg/mmol had a sensitivity of 74% and specificity of 84% for identifying those with the highest tertile of P21**^+^**KI67**^-^** senescent epithelia levels whereas the threshold 61.5 μg/mmol had a sensitivity of 82% and specificity of 67% for identifying these patients.

**Figure 6:**
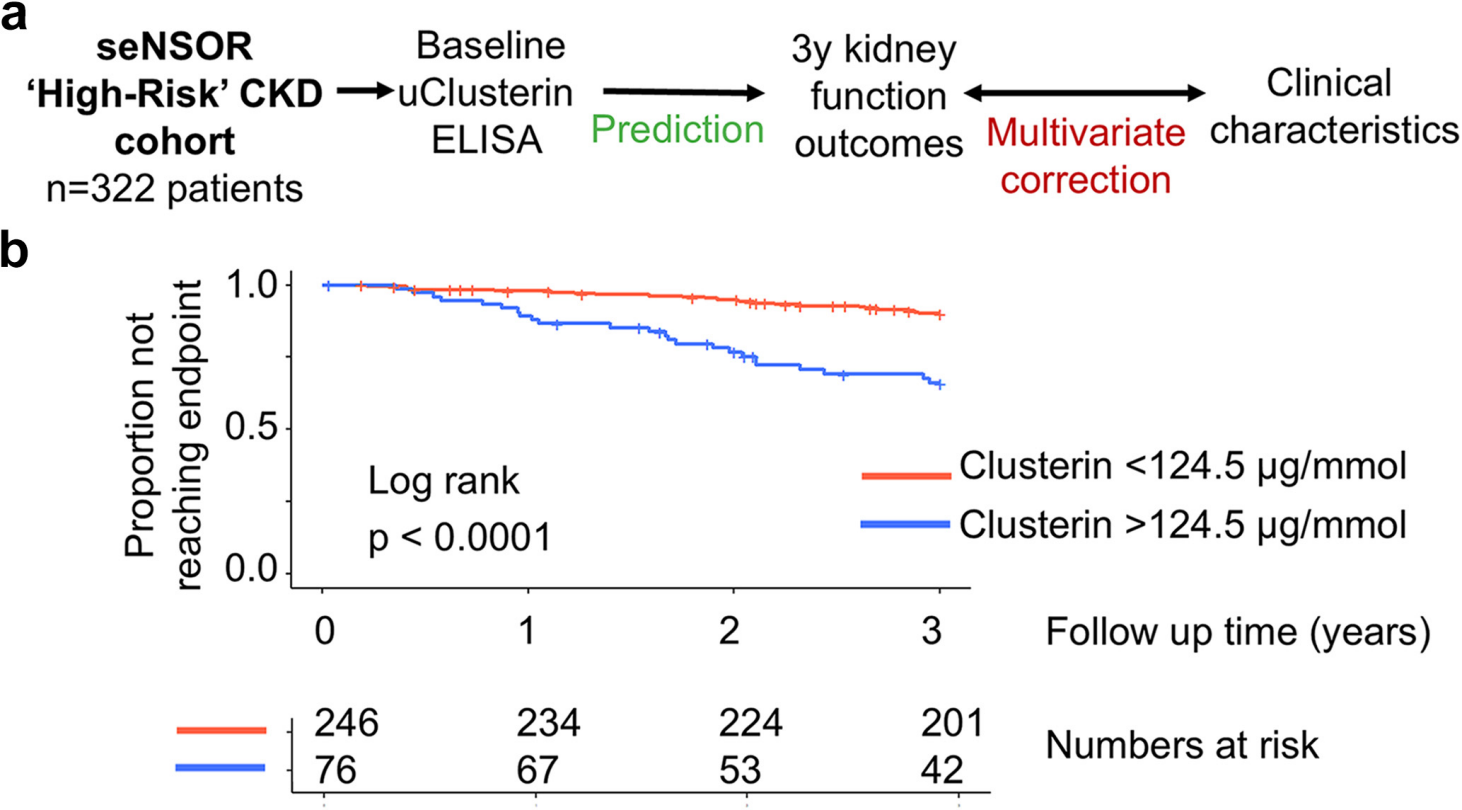
(a) Schema of analysis performed in outcome studies in human CKD (*n* = 322). (b) Proportion of patients reaching end-stage kidney disease or >40% reduction in renal function from baseline eGFR, stratified by low (<124.5 μg/mmol) or high (>124.5 μg/mmol) urinary clusterin-to-creatinine level. CKD, chronic kidney disease; eGFR, estimated glomerular filtration rate.

We tested whether uCCR predicted a decline in renal function in 322 patients at increased risk of renal progression (eGFR < 60 ml/min and/or uACR > 30 mg/mmol;S2 Figure 6a, Table 4). The composite CKD progression end point (defined as reaching end-stage kidney disease or > 40% reduction in renal function from eGFR at baseline) occurred in 47 participants (15%) during the 3-year follow-up.

**Table 4:** Baseline characteristics of the participants included in the outcome analysis

Characteristics	Outcome cohort
*n*	322
Age (yrs), median (IQR)	60.5 (47.9–69.3)
Male/Female, *n*	192/130
eGFR (ml/min per 1.73 m^2^), median (IQR)	42.1 (29.8–64.4)
SBP, median (IQR)	135 (122–148)
DBP, median (IQR)	78 (70, 84)
uACR (mg/mmol), median (IQR)	81.6 (14.5–288.8)
Ethnicity	
White, *n*	279
Black, *n*	5
Asian, *n*	12
Not recorded, *n*	26
Diagnoses, *n*	
Glomerular disease	153
Tubulointerstitial disease	44
Diabetes mellitus	29
Renovascular disease / hypertension	27
Other systemic diseases affecting kidney	7
Familial / hereditary nephropathies	10
Miscellaneous renal disorders	52

DBP, diastolic blood pressure; eGFR, estimated glomerular filtration rate; IQR, interquartile range; SBP, systolic blood pressure; uACR, urinary albumin-to-creatinine ratio.

Seventy-six participants (24%) had uCCR > 124.5 μg/mmol. These participants had a higher rate of CKD progression (24/76 [32%] vs. 23/246 [9.3%] compared with those with low uCCR, log-rank *P* < 0.001; Figure 6b). In multivariate analysis, a uCCR > 124.5 μg/mmol predicted CKD progression after adjusting for baseline eGFR, uACR, age, systolic blood pressure and sex (hazard ratio: 2.2, 95% CI: 1.07–4.66, *P* = 0.03, Table 5). Notably, the c-index of this model with uCCR was 0.762 compared with 0.746 for the same model without uCCR (Supplementary Table S3). The alternative thresholds 101 μg/mmol and 61.5 μg/mmol were also significant in univariate and multivariate models (Supplementary Tables S4 and S5). Including uCCR as a continuous variable (either untransformed or log-transformed) in multivariate models was also significant (Supplementary Tables S6 and S7).

**Table 5:** Cox proportional hazards regression model in outcome cohort including urinary clusterin-to-creatinine ratio (uCCR) as binary input above or below 124.5 μg/mmol

	Hazard ratio	*P* value	Lower CI	Upper CI
uCCR (> vs. < 124.5 μg/mmol)	2.234	0.033	1.071	4.662
Baseline eGFR (ml/min)	0.979	0.002	0.966	0.994
Ln (albumin-to-creatinine ratio)	1.254	0.033	1.007	1.562
Age (yrs)	0.976	0.038	0.955	0.998
Systolic blood pressure (mm Hg)	1.018	0.013	1.004	1.032
Sex (F vs. M)	1.022	0.945	0.552	1.893
Model C-index	0.762			

eGFR, estimated glomerular filtration rate; F, female; M, male.

## Discussion

Our data demonstrates that uCCRs represent a noninvasive surrogate for histological P21**^+^**KI67**^-^** senescent renal epithelia quantification. Clusterin was identified through unbiased urinary LC-MS/MS screening and then validated in a separate cohort using samples from patients with CKD. *CLU* (clusterin) transcripts were upregulated in hRPTECs induced to senescence *in vitro* and in proximal tubular cell epithelia expressing *CDKN1A*. Elevated uCCR predicted poor outcomes in human kidney disease independently of existing risk factors.

Although the evidence implicates senescent cells in the evolution of fibrosis and functional decline in multiple organs, there are currently no licensed biomarkers to quantify senescence in solid organs without tissue biopsy. Several phase 1 trials using medications that deplete senescent cells have shown that they are safe and well-tolerated.^13^, ^14^, ^15^ This included one study in diabetic kidney disease, which relied on serial adipose tissue and skin biopsies as less invasive but indirect surrogates for repeated kidney biopsies.^13^ We have shown that a uCCR > 124.5 μg/mmol has a high specificity for identifying patients with elevated levels of renal epithelial senescence (90%) and that these patients have poorer outcomes. Such a threshold could be used to enrich clinical trials testing senescent cell–depleting therapies with those participants most likely to benefit.

Clusterin is a glycoprotein that is purported to have antiapoptotic activity and a role in modulating the inflammatory response.^16^ It can act as a molecular chaperone and is normally secreted; however, it is released into the cytosol during cell stress.^17^

Previous *in vitro* studies using senescent hepatocytes found that they too upregulate clusterin as well as SASP markers interleukin-6 and interleukin-8.^18^^,^^19^ Using CLU short hairpin RNA to knockdown clusterin expression reduced interleukin-6 and interleukin-8, raising the possibility that clusterin can mediate SASP production.^19^

Our study has several limitations. We did not have access to matched kidney tissue and urine samples from a control group (i.e., without CKD). Notably however, our biopsy cohorts included participants with a wide range in age (19–81 years) and renal function (eGFR: 8–133 ml/min), thus, the relatively wide range in proportions of p21^+^Ki67^-^ senescent epithelia observed. We used a patient cohort recruited from secondary care nephrology services at risk of CKD progression (baseline eGFR < 60 ml/min or ACR > 30 mg/mmol) and 3-year follow-up period, limiting the generalizability of these results to those with lower risk of progression. Larger studies that include patients spanning a complete range of ethnicities and incorporating those with lower risk of CKD progression and longer follow-up will therefore be required to define the utility of uCCR in these populations. In addition, further work will be required to assess the impact of senescent cell–depleting agents on uCCR levels to determine whether uCCR could be used to track therapeutic response. The heterogeneity of senescent cells is well-recognized; we have focused on P21**^+^**KI67**^-^** renal epithelia with further work required to determine the relationship between clusterin and non-P21–dependent senescent cells. While we have demonstrated that CLU is upregulated in senescence *in vitro*, future work should include functional validation and determining the impact of clusterin silencing on senescent renal epithelial cells.

uCCR should assist in identifying patients with high predicted levels of renal epithelial senescence for clinical trials of senescent cell–depleting therapies and subsequent intensified monitoring of treatment efficacy and therapeutic response in trial participants and the wider population. In the future, its measurement has the potential to advance personalized therapy to prevent disease progression in patients with high renal senescent cell load.

## Disclosure

All authors have completed ICMJE listing any potential conflicts of interest. DPB and DAF are named on a patent application covering use of urinary Clusterin in chronic kidney disease. WY, NS, CW were employees at Nanostring (now Bruker Spatial biology) at time of study. WY and CW were holders of stock or stock options at Bruker Spatial Biology. PBM reports personal fees and/or non-financial support from GSK, Astellas, Bayer, Astra Zeneca, Boehringer Ingelheim, Vifor and Pharmacosmos. BRC received consulting fees from Argenx. DAF has received honoraria from Rejuvenon and UCB pharmaceuticals.

## Supplementary Material

Supplementary File (PDF) **Figure S1.** Correlation between urinary clusterin-to-creatinine ratio and cortical fibrosis in (A) the Edinburgh biopsy cohort (*n* = 49) and (B) the Glasgow biopsy cohort (*n* = 50). **Table S1.** Full list of proteins detected in urinary proteomic studies ranked by correlation strength of correlation with P21Ki67 epithelial senescence levels in the kidney. **Table S2.** Contingency table showing the number of cells positive and negative for P21 and clusterin in human CKD by immunofluorescence (*n* = 5). **Table S3.** Cox proportional hazards regression model in outcome cohort showing baseline variable without urinary clusterin-to-creatinine ratio (uCCR). **Table S4.** Cox proportional hazards regression model in outcome cohort including urinary clusterin-to-creatinine ratio (uCCR) as binary input > or < 101 μg/mmol. **Table S5.** Cox proportional hazards regression model in outcome cohort including urinary clusterin-to-creatinine ratio (uCCR) as binary input > or < 61.5 μg/mmol. **Table S6.** Cox proportional hazards regression model in outcome cohort including urinary clusterin-to-creatinine ratio (uCCR) as an untransformed continuous variable. **Table S7.** Cox proportional hazards regression model in outcome cohort including urinary clusterin-to-creatinine ratio (uCCR) as log-transformed continuous variable. STROBE Checklist.
