# Supplementary material for: Urinary Clusterin is a Biomarker of Renal Epithelial Senescence and Predicts Human Kidney Disease Progression
Source: Kidney Int Rep. 2025 Apr 21;10(7):2344–56. doi: 10.1016/j.ekir.2025.04.035 (PMC12266154; doi:10.1016/j.ekir.2025.04.035)
Supplement: Supplementary File (PDF) — Figure S1. Correlation between urinary clusterin-to-creatinine ratio and cortical fibrosis in (A) the Edinburgh biopsy cohort (n = 49) and (B) the Glasgow biopsy cohort (n = 50). Table S1. Full list of proteins detected in urinary proteomic studies ranked by correlation strength of correlation with P21Ki67 epithelial senescence levels in the kidney. Table S2. Contingency table showing the number of cells positive and negative for P21 and clusterin in human CKD by immunofluorescence (n = 5). Table S3. Cox proportional hazards regression model in outcome cohort showing baseline variable without urinary clusterin-to-creatinine ratio (uCCR). Table S4. Cox proportional hazards regression model in outcome cohort including urinary clusterin-to-creatinine ratio (uCCR) as binary input > or < 101 μg/mmol. Table S5. Cox proportional hazards regression model in outcome cohort including urinary clusterin-to-creatinine ratio (uCCR) as binary input > or < 61.5 μg/mmol. Table S6. Cox proportional hazards regression model in outcome cohort including urinary clusterin-to-creatinine ratio (uCCR) as an untransformed continuous variable. Table S7. Cox proportional hazards regression model in outcome cohort including urinary clusterin-to-creatinine ratio (uCCR) as log-transformed continuous variable. STROBE Checklist. [file mmc1.pdf]

Urinary Clusterin:Creatinine ratio vs cortical fibrosis

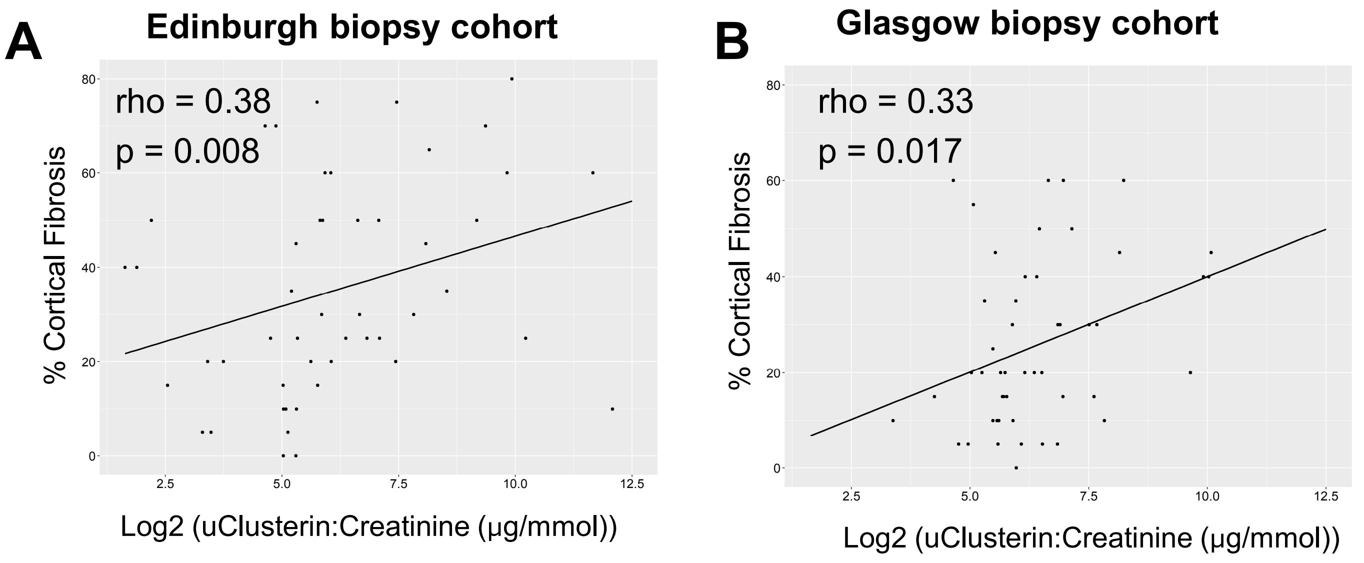

Supplemental Table 1. Full list of proteins detected in urinary proteomic studies ranked by correlation strength of correlation with P21Ki67 epithelial senescence levels in the kidney.

Gene	Protein	Rho	p value	Adjusted p value
COMP	Cartilage oligomeric matrix protein	0.580	0.00001	0.0033
PLG	Plasminogen	0.559	0.00002	0.0033
C8A	Complement component C8 alpha chain	0.546	0.00004	0.0040
APOH	Beta-2-glycoprotein 1	0.534	0.00005	0.0040
CLU	Clusterin	0.534	0.00006	0.0040
C7	Complement component C7	0.515	0.00011	0.0057
HPX	Hemopexin	0.514	0.00012	0.0057
TTR	Transthyretin	0.500	0.00019	0.0076
EFEMP1	EGF-containing fibulin-like extracellular matrix protein 1	0.494	0.00023	0.0076
PROS1	Vitamin K-dependent protein S	0.491	0.00025	0.0076
LUM	Lumican	0.489	0.00027	0.0076
FGA	Fibrinogen alpha chain	0.488	0.00028	0.0076
CTSD	Cathepsin D	0.486	0.00030	0.0076
SERPINA3	Alpha-1-antichymotrypsin	0.482	0.00035	0.0079
SERPING1	Plasma protease C1 inhibitor	0.480	0.00037	0.0079
C4A	Complement C4-A	0.479	0.00038	0.0079
C8G	Complement component C8 gamma chain	0.471	0.00049	0.0090
CFHR1	Complement factor H-related protein 1	0.471	0.00049	0.0090
SERPINC1	Antithrombin-III	0.464	0.00061	0.0099
IGLV3_21	NA	0.462	0.00063	0.0099
CRISP3	Cysteine-rich secretory protein 3 (Fragment)	0.462	0.00064	0.0099
F2	Prothrombin	0.461	0.00066	0.0099
GC_297	NA	0.457	0.00075	0.0107
GC_62	NA	0.455	0.00080	0.0107
C3	Complement C3	0.454	0.00081	0.0107
SERPINF1	Pigment epithelium-derived factor	0.451	0.00089	0.0113
GM2A	Ganglioside GM2 activator	0.445	0.00106	0.0128
ITIH4	ITIH4 protein	0.442	0.00115	0.0128
C9	Complement component C9	0.441	0.00119	0.0128
IGLC3	Ig lambda-3 chain C regions (Fragment)	0.440	0.00125	0.0128
HSPG2	Basement membrane-specific heparan sulfate proteoglycan core protein	0.439	0.00125	0.0128
C4BPA	C4b-binding protein alpha chain	0.438	0.00130	0.0128
AMBP	Protein AMBP	0.438	0.00130	0.0128
APOA1	Apolipoprotein A-I	0.437	0.00135	0.0128
PRG4	Proteoglycan 4	0.435	0.00142	0.0128
RNASE1	Ribonuclease pancreatic	0.435	0.00143	0.0128

IGLL5	Immunoglobulin lambda-like polypeptide 5	0.435	0.00143	0.0128
CFHR2	Complement factor H-related protein 2	0.432	0.00154	0.0132
PGLYRP2	N-acetylmuramoyl-L-alanine amidase	0.430	0.00165	0.0132
LDHB	L-lactate dehydrogenase B chain	0.430	0.00165	0.0132
RBP4	Retinol-binding protein 4	0.429	0.00170	0.0132
IGHA1	Ig alpha-1 chain C region	0.427	0.00180	0.0132
IGFBP6	Insulin-like growth factor-binding protein 6	0.427	0.00180	0.0132
WISP2	WNT1-inducible-signaling pathway protein 2	0.426	0.00183	0.0132
ORM1	Alpha-1-acid glycoprotein 1	0.426	0.00183	0.0132
ITIH2	Inter-alpha-trypsin inhibitor heavy chain H2	0.426	0.00184	0.0132
SLURP1	Secreted Ly-6/uPAR-related protein 1	0.421	0.00210	0.0148
C2	Complement C2	0.420	0.00214	0.0148
CFD	Complement factor D	0.416	0.00240	0.0162
APOD	Apolipoprotein D (Fragment)	0.413	0.00258	0.0171
FN1	Fibronectin	0.412	0.00267	0.0173
AGT	Angiotensinogen	0.409	0.00288	0.0183
IGHG1	Ig gamma-1 chain C region	0.407	0.00300	0.0187
LY6D	Lymphocyte antigen 6D	0.403	0.00333	0.0204
FBN1	Fibrillin-1	0.402	0.00348	0.0209
AHSG	Alpha-2-HS-glycoprotein	0.397	0.00388	0.0229
HRG	Histidine-rich glycoprotein	0.394	0.00424	0.0246
VTN	Vitronectin	0.392	0.00446	0.0251
UBA52	Ubiquitin-60S ribosomal protein L40 (Fragment)	0.392	0.00447	0.0251
IGFBP4	Insulin-like growth factor-binding protein 4	0.390	0.00469	0.0257
C8B	Complement component C8 beta chain	0.389	0.00481	0.0257
MSMB	Beta-microseminoprotein	0.389	0.00482	0.0257
IGLV2_11	NA	0.386	0.00512	0.0269
CP	Ceruloplasmin	0.382	0.00564	0.0289
CD5L	CD5 antigen-like	0.381	0.00576	0.0289
ITIH1	Inter-alpha-trypsin inhibitor heavy chain H1	0.381	0.00577	0.0289
NPC2	Epididymal secretory protein E1 (Fragment)	0.378	0.00619	0.0302
ORM2	Alpha-1-acid glycoprotein 2	0.378	0.00620	0.0302
GSN	Gelsolin	0.377	0.00634	0.0304
CPN2	Carboxypeptidase N subunit 2	0.375	0.00671	0.0317
PRSS2	Trypsin-2	0.374	0.00694	0.0324
CST6	Cystatin-M	0.372	0.00720	0.0331
SH3BGRL3	SH3 domain-binding glutamic acid-rich-like protein 3	0.371	0.00738	0.0335
FGB	Fibrinogen beta chain	0.365	0.00840	0.0375
TNFR1B	Soluble TNFR1B variant 1	0.365	0.00849	0.0375
PIGR	Polymeric immunoglobulin receptor	0.360	0.00939	0.0409
C5	Complement C5	0.359	0.00958	0.0412
VCAM1	Vascular cell adhesion protein 1	0.356	0.01035	0.0439
FETUB	Fetuin-B	0.354	0.01092	0.0458
NBL1	Neuroblastoma suppressor of tumorigenicity 1	0.350	0.01185	0.0490

CFH	Complement factor H	0.349	0.01208	0.0494
CD59	CD59 glycoprotein	0.347	0.01264	0.0506
ENO1	Alpha-enolase	0.346	0.01279	0.0506
PFN1	Profilin-1	0.346	0.01284	0.0506
LY6H	Lymphocyte antigen 6H	0.344	0.01345	0.0522
IGLC7	Ig lambda-7 chain C region	0.344	0.01356	0.0522
SERPINF2	Alpha-2-antiplasmin	0.343	0.01388	0.0526
KNG1	Kininogen-1	0.342	0.01398	0.0526
IGHG4	Ig gamma-4 chain C region (Fragment)	0.341	0.01426	0.0530
AMY2A	Pancreatic alpha-amylase	0.339	0.01501	0.0549
YWHAB	14-3-3 protein beta/alpha	0.339	0.01510	0.0549
IGHV3_72	NA	0.338	0.01527	0.0549
MT2A	Metallothionein-2	0.337	0.01550	0.0552
CD14	Monocyte differentiation antigen CD14	0.336	0.01607	0.0562
CST3	Cystatin-C	0.335	0.01623	0.0562
CA1	Carbonic anhydrase 1	0.335	0.01629	0.0562
APOA4	Apolipoprotein A-IV	0.334	0.01666	0.0569
IGHA2	Ig alpha-2 chain C region (Fragment)	0.332	0.01739	0.0587
APOA2	Apolipoprotein A-II (Fragment)	0.331	0.01779	0.0595
PTGDS	Prostaglandin-H2 D-isomerase	0.330	0.01819	0.0602
THY1	Thy-1 membrane glycoprotein	0.328	0.01863	0.0611
C1RL	Complement C1r subcomponent-like protein	0.328	0.01884	0.0611
EFNA1	Ephrin-A1	0.326	0.01958	0.0623
AZGP1	Zinc-alpha-2-glycoprotein	0.326	0.01958	0.0623
SOD1	Superoxide dismutase [Cu-Zn]	0.325	0.02012	0.0634
ENDOD1	Endonuclease domain-containing 1 protein	0.323	0.02064	0.0640
ECM1	Extracellular matrix protein 1	0.323	0.02068	0.0640
IGKV2D_28	NA	0.323	0.02087	0.0640
VSIG4	V-set and immunoglobulin domain-containing protein 4	0.318	0.02288	0.0695
F12	Coagulation factor XII	0.314	0.02465	0.0742
FBLN1	Fibulin-1	0.314	0.02496	0.0744
AGRN	Agrin	0.312	0.02572	0.0760
IGFBP1	Insulin-like growth factor-binding protein 1	0.310	0.02701	0.0791
HP	Haptoglobin	0.309	0.02758	0.0801
LRG1	Leucine-rich alpha-2-glycoprotein	0.308	0.02811	0.0809
ELANE	Neutrophil elastase	0.307	0.02860	0.0816
CHIT1	Chitotriosidase-1	0.306	0.02919	0.0823
JCHAIN	Immunoglobulin J chain (Fragment)	0.305	0.02935	0.0823
APOE	Apolipoprotein E	0.305	0.02972	0.0827
YWHAZ	14-3-3 protein zeta/delta (Fragment)	0.302	0.03134	0.0865
PEBP4	Phosphatidylethanolamine-binding protein 4	0.300	0.03267	0.0894
C6	Complement component C6	0.290	0.03877	0.1052
PROZ	Vitamin K-dependent protein Z	0.286	0.04188	0.1123
TF	Serotransferrin	0.286	0.04226	0.1123

CDH1	Cadherin-1	0.285	0.04267	0.1123
IGFBP7	Insulin-like growth factor-binding protein 7	0.285	0.04274	0.1123
CTSB	Cathepsin B	0.283	0.04420	0.1152
APOL1	Apolipoprotein L1	0.283	0.04456	0.1152
ESAM	Endothelial cell-selective adhesion molecule	0.281	0.04556	0.1169
FABP4	Fatty acid-binding protein, adipocyte	0.279	0.04733	0.1205
LGALS3BP	Galectin-3-binding protein	0.273	0.05289	0.1336
IGFBP3	Insulin-like growth factor-binding protein 3 (Fragment)	0.272	0.05348	0.1341
KRT81	Keratin, type II cuticular Hb1	-0.271	0.05407	0.1346
A1BG	Alpha-1B-glycoprotein	0.270	0.05539	0.1368
COL18A1	Collagen alpha-1(XVIII) chain (Fragment)	0.269	0.05607	0.1375
FGG	Fibrinogen gamma chain	0.266	0.05896	0.1430
MMP9	Matrix metalloproteinase-9	0.266	0.05918	0.1430
A2M	Alpha-2-macroglobulin	0.264	0.06077	0.1458
ARSA	Arylsulfatase A	0.263	0.06206	0.1478
MST1L	Putative macrophage stimulating 1-like protein	0.263	0.06251	0.1478
MYH9	Myosin-9	0.262	0.06326	0.1485
LCN2	Neutrophil gelatinase-associated lipocalin	0.261	0.06472	0.1509
DEFB1	Beta-defensin 1	0.260	0.06563	0.1519
AFM	Afamin	0.255	0.07060	0.1623
CORO1A	Coronin-1A	0.254	0.07165	0.1636
SIRPA	Tyrosine-protein phosphatase non-receptor type substrate 1	0.253	0.07279	0.1650
LYZ	Lysozyme C	0.251	0.07526	0.1695
MPO	Myeloperoxidase	0.251	0.07591	0.1698
LTF	Lactotransferrin	0.250	0.07701	0.1711
ALDOA	Fructose-bisphosphate aldolase	0.249	0.07851	0.1733
CD248	Endosialin	0.248	0.07924	0.1737
LTBP2	Latent-transforming growth factor beta-binding protein 2	0.246	0.08130	0.1770
SPP1	Osteopontin	0.242	0.08748	0.1893
TFF2	Trefoil factor 2	-0.240	0.08932	0.1920
ENPEP	Glutamyl aminopeptidase	0.238	0.09321	0.1991
SIRPB1	Signal-regulatory protein beta-1	0.236	0.09530	0.2022
PRDX2	Peroxiredoxin-2	0.235	0.09672	0.2039
IGKV6_21	NA	0.233	0.09922	0.2079
PGLYRP1	Peptidoglycan recognition protein 1	0.231	0.10285	0.2141
RNASET2	Ribonuclease T2	0.230	0.10467	0.2160
HLA_B	NA	0.230	0.10505	0.2160
KLK6	Kallikrein-6	0.229	0.10678	0.2182
CALR	Calreticulin	0.228	0.10807	0.2195
HABP2	Hyaluronan-binding protein 2	0.226	0.11054	0.2231
COTL1	Coactosin-like protein	0.226	0.11139	0.2235
PON1	Serum paraoxonase/arylesterase 1	0.224	0.11335	0.2260

DSC1	Desmocollin-1	0.223	0.11493	0.2278
ACTB	Actin, cytoplasmic 1	0.223	0.11652	0.2296
ITIH3	Inter-alpha-trypsin inhibitor heavy chain H3	0.219	0.12337	0.2416
IGFBP5	Insulin-like growth factor-binding protein 5 (Fragment)	0.217	0.12660	0.2465
KLKB1	Plasma kallikrein (Fragment)	0.215	0.12927	0.2502
RNASE2	Non-secretory ribonuclease	0.214	0.13170	0.2523
LYVE1	Lymphatic vessel endothelial hyaluronic acid receptor 1	0.214	0.13185	0.2523
VMO1	Vitelline membrane outer layer protein 1 homolog	0.213	0.13290	0.2528
LYNX1_192	NA	0.211	0.13747	0.2600
PI16	Peptidase inhibitor 16	0.210	0.13991	0.2631
HSPA1A	Heat shock 70 kDa protein 1A	0.208	0.14303	0.2675
RNASE4	Ribonuclease 4	0.207	0.14559	0.2702
SECTM1	Secreted and transmembrane protein 1 (Fragment)	0.206	0.14613	0.2702
EFNB1	Ephrin-B1	0.206	0.14777	0.2717
LMAN2	Vesicular integral-membrane protein VIP36	0.199	0.16249	0.2972
CD44	CD44 antigen (Fragment)	0.198	0.16406	0.2984
FAM3C	Protein FAM3C	0.193	0.17556	0.3175
SERPINA5	Plasma serine protease inhibitor	0.191	0.17972	0.3233
CFI	Complement factor I	0.185	0.19488	0.3487
F13B	Coagulation factor XIII B chain	0.184	0.19666	0.3500
B2M	Beta-2-microglobulin	0.183	0.19847	0.3513
MGAM	Maltase-glucoamylase, intestinal (Fragment)	0.181	0.20252	0.3558
LPA	Apolipoprotein(a)	0.181	0.20348	0.3558
RETN	Resistin	0.180	0.20499	0.3558
CHGA	Chromogranin-A	-0.180	0.20531	0.3558
MFAP5	Microfibrillar-associated protein 5 (Fragment)	0.178	0.21237	0.3644
PVRL2	Nectin-2	0.177	0.21304	0.3644
C1QB	Complement C1q subcomponent subunit B (Fragment)	0.177	0.21434	0.3644
LTBP1	Latent-transforming growth factor beta-binding protein 1	0.176	0.21639	0.3644
SERPIND1	Heparin cofactor 2	0.176	0.21674	0.3644
REG1A	Lithostathine-1-alpha	0.176	0.21687	0.3644
C1S	Complement C1s subcomponent	0.175	0.21815	0.3647
GKN1	Gastrokine-1	0.174	0.22147	0.3684
CD40	CD40 antigen (TNF receptor superfamily member 5), isoform CRA_c	0.173	0.22406	0.3708
ANPEP	Aminopeptidase N	0.173	0.22515	0.3708
HIST1H2AG	Histone H2A type 1	0.169	0.23694	0.3883
COL15A1	Collagen alpha-1(XV) chain	0.165	0.24744	0.4035
PSAP	Prosaposin	0.164	0.25096	0.4053
PAPPA2	Pappalysin-2	0.163	0.25193	0.4053

ICAM1	Intercellular adhesion molecule 1	0.163	0.25226	0.4053
ROBO4	Roundabout homolog 4	0.163	0.25390	0.4060
ATRN	Attractin	0.162	0.25609	0.4069
F10	Coagulation factor X	0.162	0.25692	0.4069
FABP1	Fatty acid-binding protein, liver	0.159	0.26646	0.4200
SCUBE2	Signal peptide, CUB and EGF-like domain-containing protein 2	0.158	0.26922	0.4223
C1R	Complement C1r subcomponent	-0.157	0.27229	0.4251
IGLV2_14	NA	0.153	0.28334	0.4403
BTN2A1	Butyrophilin subfamily 2 member A1	-0.152	0.28743	0.4446
MEP1A	Metalloendopeptidase	0.150	0.29270	0.4506
SPINK5	Serine protease inhibitor Kazal-type 5	0.148	0.30070	0.4601
MASP2	Mannan-binding lectin serine protease 2	0.148	0.30161	0.4601
SCGB1A1	Uteroglobin	0.146	0.30643	0.4636
CFP	Properdin	0.146	0.30738	0.4636
SERPINA4	Kallistatin	-0.146	0.30811	0.4636
HGFAC	Hepatocyte growth factor activator	0.143	0.31776	0.4759
MB	Myoglobin (Fragment)	0.141	0.32212	0.4803
ART3	NAD(P)(+)--arginine ADP-ribosyltransferase (Fragment)	0.140	0.32889	0.4882
ANXA2	Annexin (Fragment)	0.137	0.33607	0.4966
F9	Coagulation factor IX	0.136	0.34027	0.4991
FLNA	Filamin-A	0.136	0.34080	0.4991
CDH6	Cadherin-6	-0.132	0.35410	0.5163
PRNP	Major prion protein (Fragment)	0.130	0.36194	0.5255
DSC3	Desmocollin-3	-0.129	0.36802	0.5319
SERPINA7	Thyroxine-binding globulin	0.126	0.37737	0.5431
TWSG1	Twisted gastrulation protein homolog 1	0.124	0.38780	0.5557
CAPG	Macrophage-capping protein (Fragment)	-0.121	0.39675	0.5641
COL6A1	Collagen alpha-1(VI) chain	0.121	0.39804	0.5641
PSCA	Prostate stem cell antigen	0.121	0.39882	0.5641
PPBP	Platelet basic protein	0.120	0.40299	0.5676
CDH13	Cadherin-13	0.119	0.40519	0.5683
HYOU1	Hypoxia up-regulated protein 1	0.118	0.40914	0.5714
CTGF	Connective tissue growth factor	0.117	0.41205	0.5725
TXNDC5	Thioredoxin domain-containing protein 5	0.117	0.41371	0.5725
TXN	Thioredoxin	0.117	0.41511	0.5725
GNS	N-acetylglucosamine-6-sulfatase	0.115	0.42045	0.5775
CAMP	Cathelicidin antimicrobial peptide	0.114	0.42709	0.5814
HIST1H1E	Histone H1.4	0.113	0.42849	0.5814
DKK3	Dickkopf-related protein 3	0.113	0.42857	0.5814
SPARCL1	SPARC-like protein 1	0.110	0.44250	0.5973
PRSS1	Protease serine 1	-0.110	0.44394	0.5973
TNFRSF1A	Tumor necrosis factor receptor superfamily member 1A (Fragment)	0.109	0.44715	0.5992

WFDC2	WAP four-disulfide core domain protein 2	0.108	0.44926	0.5996
ANXA1	Annexin A1	0.107	0.45538	0.6046
CD27	CD27 antigen	0.106	0.45845	0.6046
CCL14	C-C motif chemokine 14	0.106	0.45845	0.6046
PEBP1	Phosphatidylethanolamine-binding protein 1	-0.104	0.46697	0.6124
IGFBP2	Insulin-like growth factor-binding protein 2	0.104	0.46810	0.6124
KRT9	Keratin, type I cytoskeletal 9	0.102	0.47676	0.6193
LRP2	Low-density lipoprotein receptor-related protein 2	0.102	0.47711	0.6193
HBB	Hemoglobin subunit beta	0.101	0.47912	0.6195
ASAH1	Acid ceramidase	0.096	0.50248	0.6446
SERPINB1	Leukocyte elastase inhibitor	-0.095	0.50534	0.6446
PCOLCE	Procollagen C-endopeptidase enhancer 1	0.095	0.50633	0.6446
APOB	Apolipoprotein B-100	0.095	0.50634	0.6446
COL5A1	Collagen alpha-1(V) chain	0.094	0.51127	0.6475
CD300A	CMRF35-like molecule 8	0.094	0.51255	0.6475
SEMG1	Semenogelin-1	0.091	0.52673	0.6626
PGA4	Pepsin A-4	0.090	0.52851	0.6626
TPI1	Triosephosphate isomerase	0.089	0.53391	0.6669
IGHG3	Ig gamma-3 chain C region	0.089	0.53642	0.6675
MCAM	Cell surface glycoprotein MUC18	0.088	0.53890	0.6681
LAMP1	Lysosome-associated membrane glycoprotein 1	0.086	0.54860	0.6776
CD300LG	CMRF35-like molecule 9	0.081	0.57250	0.7045
LOX	Protein-lysine 6-oxidase	0.079	0.58115	0.7125
AHCY	Adenosylhomocysteinase	0.076	0.59598	0.7279
CDH11	Cadherin-11	-0.075	0.60242	0.7331
FLG	Filaggrin	-0.072	0.61342	0.7428
PILRA	Paired immunoglobulin-like type 2 receptor alpha (Fragment)	0.072	0.61491	0.7428
FCGR3B	Low affinity immunoglobulin gamma Fc region receptor III-B	0.071	0.61889	0.7449
HIST1H4A	Histone H4	0.071	0.62168	0.7456
SH3BGRL	SH3 domain-binding glutamic acid-rich-like protein	0.069	0.62809	0.7505
RNASE6	Ribonuclease K6	0.068	0.63469	0.7557
SERPINB3	SCCA1/SCCA2 fusion protein	-0.065	0.64963	0.7679
FSTL1	Follistatin-related protein 1 (Fragment)	0.064	0.65377	0.7679
TKT	Transketolase	-0.064	0.65393	0.7679
NID1	Nidogen-1	-0.064	0.65419	0.7679
CD55	Complement decay-accelerating factor	0.062	0.66380	0.7764
LCP1	Plastin-2	-0.058	0.68740	0.7951
VWF	von Willebrand factor	0.058	0.68765	0.7951
HMCN1	Hemicentin-1 (Fragment)	0.058	0.68838	0.7951
CADM3	Cell adhesion molecule 3 (Fragment)	0.057	0.68939	0.7951
KRT16	Keratin, type I cytoskeletal 16	-0.054	0.70464	0.8081

IGF2	Insulin-like growth factor II	0.054	0.70557	0.8081
COL6A3	Collagen alpha-3(VI) chain	0.053	0.71445	0.8130
CRNN	Cornulin	0.052	0.71474	0.8130
EGF	Pro-epidermal growth factor	-0.051	0.71968	0.8131
PVR	Poliovirus receptor	0.051	0.71973	0.8131
CHI3L1	Chitinase-3-like protein 1	-0.050	0.72989	0.8218
KRT5	Keratin, type II cytoskeletal 5	-0.048	0.73821	0.8283
HBA1	Hemoglobin subunit alpha	0.046	0.74919	0.8364
SERPINA6	Corticosteroid-binding globulin	0.045	0.75254	0.8364
S100A9	Protein S100-A9	0.045	0.75301	0.8364
EPHA1	Ephrin type-A receptor 1	0.044	0.76057	0.8420
HIST1H2BE	Histone H2B	0.043	0.76541	0.8423
KRT2	Keratin, type II cytoskeletal 2 epidermal	0.043	0.76599	0.8423
FOLR1	Folate receptor alpha	-0.042	0.77123	0.8453
UMOD	Uromodulin	-0.041	0.77420	0.8457
KRT1	Keratin, type II cytoskeletal 1	0.040	0.77855	0.8477
KLK3	Prostate-specific antigen (Fragment)	0.038	0.79063	0.8580
FLNC	Filamin-C	0.037	0.79675	0.8618
CTSZ	Cathepsin Z	0.036	0.80138	0.8640
KRT14	Keratin, type I cytoskeletal 14	-0.030	0.83274	0.8931
DAG1	Dystroglycan	-0.030	0.83377	0.8931
CEACAM8	Carcinoembryonic antigen-related cell adhesion molecule 8	0.029	0.83840	0.8952
KPRP	Keratinocyte proline-rich protein	0.028	0.84704	0.9015
MSN	Moesin	0.026	0.85879	0.9111
SOD3	Extracellular superoxide dismutase [Cu-Zn]	-0.022	0.87566	0.9239
CRHBP	Corticotropin-releasing factor-binding protein	0.022	0.87749	0.9239
KRT10	Keratin, type I cytoskeletal 10	-0.022	0.87926	0.9239
ALB	Serum albumin	0.021	0.88374	0.9249
KRT6A	Keratin, type II cytoskeletal 6A	-0.021	0.88573	0.9249
LBP	Lipopolysaccharide-binding protein	0.019	0.89469	0.9301
NEGR1	Neuronal growth regulator 1	0.018	0.89761	0.9301
KLK1	Kallikrein-1	-0.018	0.89918	0.9301
FLG2	Filaggrin-2	0.017	0.90355	0.9317
HRNR	Hornerin	-0.015	0.91517	0.9408
TPM3	Tropomyosin alpha-3 chain	0.014	0.92015	0.9429
TALDO1	Transaldolase	0.013	0.92676	0.9460
FABP5	Fatty acid-binding protein, epidermal	-0.012	0.93069	0.9460
LYNX1_264	NA	0.012	0.93169	0.9460
FOLR2	Folate receptor beta	-0.010	0.94397	0.9555
SUMF2	Sulfatase-modifying factor 2	-0.008	0.95471	0.9626
GRN	Granulins	-0.008	0.95677	0.9626
IFI30	Gamma-interferon-inducible lysosomal thiol reductase	0.006	0.96481	0.9677
FCGBP	IgGFc-binding protein	0.004	0.97838	0.9784

Supplemental Table 2. Contingency table showing number of cells positive and negative for P21 and Clusterin in human CKD by immunofluorescence (n=5).

	p21 positive cells	p21 negative cells
Clusterin positive cells	3053	52566
Clusterin negative cells	4609	169800

Supplemental Table 3. Cox proportional hazards regression model in outcome cohort showing baseline variable without urinary clusterin:creatinine ratio (uCCR).

	**Hazard ratio**	**p value**	**Lower CI**	**Upper CI**
Baseline eGFR (mls/min)	0.976	<0.001	0.962	0.990
Ln (Albumin Creatinine Ratio)	1.447	<0.001	1.197	1.748
Age (years)	0.977	0.04	0.955	0.999
Systolic Blood Pressure (mmHg)	1.02	0.006	1.006	1.033
Sex (F vs M)	1.059	0.855	0.573	1.956
Model C-index	0.746			

Supplemental Table 4. Cox proportional hazards regression model in outcome cohort including urinary clusterin:creatinine ratio (uCCR) as binary input above or below 101µg/mmol.

	**Hazard ratio**	**p value**	**Lower CI**	**Upper CI**
Clusterin (above vs below 101µg/mmol)	2.145	0.045	1.015	4.533
Baseline eGFR (mls/min)	0.979	0.002	0.965	0.993
Ln (Albumin Creatinine Ratio)	1.251	0.038	1.0003	1.566
Age (years)	0.977	0.042	0.956	0.999
Systolic Blood Pressure (mmHg)	1.018	0.010	1.004	1.033
Sex (F vs M)	1.030	0.924	0.557	1.906
Model C-index	0.761			

Supplemental Table 5. Cox proportional hazards regression model in outcome cohort including urinary clusterin:creatinine ratio (uCCR) as binary input above or below 61.5μg/mmol.

	**Hazard ratio**	**p value**	**Lower CI**	**Upper CI**
Clusterin (above vs below 61.5μg/mmol)	2.255	0.032	1.058	4.808
Baseline eGFR (mls/min)	0.977	0.001	0.964	0.991
Ln (Albumin Creatinine Ratio)	1.258	0.028	1.013	1.563
Age (years)	0.975	0.027	0.953	0.997
Systolic Blood Pressure (mmHg)	1.020	0.007	1.005	1.034
Sex (F vs M)	0.970	0.924	0.522	1.804
Model C-index	0.753			

Supplemental Table 6. Cox proportional hazards regression model in outcome cohort including urinary clusterin:creatinine ratio (uCCR) as an untransformed continuous variable.

	**Hazard ratio**	**p value**	**Lower CI**	**Upper CI**
Clusterin:Creatinine ratio per 100 μg/mmol	1.060	0.011	1.022	1.099
Baseline eGFR (mls/min)	0.977	0.001	0.964	0.991
Ln (Albumin Creatinine Ratio)	1.321	0.003	1.090	1.602
Age (years)	0.974	0.024	0.952	0.996
Systolic Blood Pressure (mmHg)	1.020	0.005	1.006	1.033
Sex (F vs M)	1.102	0.76	0.593	2.049
Model C-index	0.757			

Supplemental Table 7. Cox proportional hazards regression model in outcome cohort including urinary clusterin:creatinine ratio (uCCR) as log transformed continuous variable.

	**Hazard ratio**	**p value**	**Lower CI**	**Upper CI**
Log_2 (Clusterin:Creatinine)	1.328	0.011	1.075	1.640
Baseline eGFR (mls/min)	0.980	0.004	0.966	0.995
Ln (Albumin Creatinine Ratio)	1.154	0.222	0.910	1.464
Age (years)	0.975	0.028	0.954	0.997
Systolic Blood Pressure (mmHg)	1.018	0.01	1.004	1.032
Sex (F vs M)	1.067	0.839	0.573	1.987
Model C-index	0.762			

STROBE Statement—checklist of items that should be included in reports of observational studies

	Item No.	Recommendation	Page No.	Relevant text from manuscript
**Title and abstract**	1	(*a*) Indicate the study's design with a commonly used term in the title or the abstract	1	"Urinary Clusterin is a biomarker of renal epithelial senescence and predicts human kidney disease progression"
		(*b*) Provide in the abstract an informative and balanced summary of what was done and what was found	2.	Included on page 2.
**Introduction**				
Background/rationale	2	Explain the scientific background and rationale for the investigation being reported	2 – Introduction paragraphs 1 and 2	
Objectives	3	State specific objectives, including any prespecified hypotheses	2	"Here we addressed the hypothesis that p21+Ki67- senescent renal epithelia could be quantified non-invasively by detecting their selectively secreted proteins in human urine samples."
**Methods**				
Study design	4	Present key elements of study design early in the paper	Inlcuded in final paragraph of introduction (page 3) then expanded in methods p 4 to p 11	"Here, we addressed the hypothesis that P21+KI67- senescent renal epithelia could be quantified non-invasively by detecting their selectively secreted proteins in human urine samples and then determined if these non-invasive surrogates of

				senescence, predicted outcomes in patients with CKD.”
Setting	5	Describe the setting, locations, and relevant dates, including periods of recruitment, exposure, follow-up, and data collection	Page 4.	Paragraph titled “seNSOR biobank recruitment”.
Participants	6	(*a*) *Cohort study*—Give the eligibility criteria, and the sources and methods of selection of participants. Describe methods of follow-up<br>*Case-control study*—Give the eligibility criteria, and the sources and methods of case ascertainment and control selection. Give the rationale for the choice of cases and controls<br>*Cross-sectional study*—Give the eligibility criteria, and the sources and methods of selection of participants	Pages 4 and 5. Summarized in figure 1.	Sections “Patient selection for biopsy cohorts” and “Patient selection for outcome analysis”
		(*b*) *Cohort study*—For matched studies, give matching criteria and number of exposed and unexposed<br>*Case-control study*—For matched studies, give matching criteria and the number of controls per case	N/A	
Variables	7	Clearly define all outcomes, exposures, predictors, potential confounders, and effect modifiers. Give diagnostic criteria, if applicable	Page 11	For the outcome analysis, CKD progression was defined as reaching ESKD (starting renal replacement therapy (RRT) or maintaining an eGFR <15mls/min for >90 days) or >40% reduction in renal function from eGFR at baseline (maintained for >90 days).
Data sources/ measurement	8*	For each variable of interest, give sources of data and details of methods of assessment (measurement). Describe comparability of assessment methods if there is more than one group	Methods -pages 4 to 11.	
Bias	9	Describe any efforts to address potential sources of bias	All patients meeting eligibility criteria in seNSOR cohort with samples	

			available included. Outcome models adjusted for known risk factors of CKD progression.	
Study size	10	Explain how the study size was arrived at	Methods -pages 4 and 5.	Summarised in Figure 1.

Continued on next page

Quantitative variables	11	Explain how quantitative variables were handled in the analyses. If applicable, describe which groupings were chosen and why	Results, pages 12 to 14. Clusterin threshold generation explained in page 14.	"Next, we explored the optimal uCCR required to identify patients with high levels of P21+KI67- senescent epithelia. On the receiver operating characteristic (ROC), the area under the curve (AUC) was 0.81 (95% C.I. 0.71-0.91, $p<0.001$ compared to null AUC of 0.5) (Figure 5). By prioritising high specificity over high sensitivity, a threshold of 124.5 µg uClusterin/mmol Creatinine was selected. This corresponded to a sensitivity of 68% and specificity of 90% for identifying patients in the highest tertile of P21+KI67- senescent epithelia proportions. Alternative threshold values were also considered; uCCR levels >101 µg/mmol had a sensitivity of 74% and specificity of 84% for identifying those with the highest tertile of P21+KI67- senescent epithelia levels whilst the threshold 61.5 µg/mmol had a sensitivity of 82% and specificity of 67% for identifying these patients
Statistical methods	12	(*a*) Describe all statistical methods, including those used to control for confounding	Methods - page 10 and 11.	See "statistical tests section".
		(*b*) Describe any methods used to examine subgroups and interactions	N/A	

		(*c*) Explain how missing data were addressed	N/A	
		(*d*) *Cohort study*—If applicable, explain how loss to follow-up was addressed<br>*Case-control study*—If applicable, explain how matching of cases and controls was addressed<br>*Cross-sectional study*—If applicable, describe analytical methods taking account of sampling strategy	Methods - page 11.	"Death was treated as a censoring event."
		(*e*) Describe any sensitivity analyses	N/A	
**Results**				
Participants	13*	(a) Report numbers of individuals at each stage of study—eg numbers potentially eligible, examined for eligibility, confirmed eligible, included in the study, completing follow-up, and analysed	Methods pages 4 to 11.	Summarised in Figure 1
		(b) Give reasons for non-participation at each stage	Methods - pages 4 and 5.	Summarised in Figure 1.
		(c) Consider use of a flow diagram	Included in figures.	See Figure 1.
Descriptive data	14*	(a) Give characteristics of study participants (eg demographic, clinical, social) and information on exposures and potential confounders	Included.	See table 1 and table 4.
		(b) Indicate number of participants with missing data for each variable of interest	NA	
		(c) *Cohort study*—Summarise follow-up time (eg, average and total amount)	14	"The composite CKD progression endpoint … occurred in 47 (15%) participants during the 3-year follow-up."
Outcome data	15*	*Cohort study*—Report numbers of outcome events or summary measures over time		
		Case-control study—Report numbers in each exposure category, or summary measures of exposure		
		Cross-sectional study—Report numbers of outcome events or summary measures	*14*	"The composite CKD progression endpoint … occurred in 47 (15%) participants during the 3-year follow-up."
Main results	16	(*a*) Give unadjusted estimates and, if applicable, confounder-adjusted estimates and their precision (eg, 95% confidence interval). Make clear which confounders were adjusted for and why they were included	Pages 12 to 14.	Summarised in Figure 2 to 6, table 5 and supplemental tables 3 to 7.

	(*b*) Report category boundaries when continuous variables were categorized	14	"…a threshold of 124.5 µg Clusterin/mmol Creatinine was selected."
	(*c*) If relevant, consider translating estimates of relative risk into absolute risk for a meaningful time period		

Continued on next page

Other analyses	17	Report other analyses done—eg analyses of subgroups and interactions, and sensitivity analyses	N/A	
**Discussion**				
Key results	18	Summarise key results with reference to study objectives	15	
Limitations	19	Discuss limitations of the study, taking into account sources of potential bias or imprecision. Discuss both direction and magnitude of any potential bias	16	Our study has several limitations. We did not have access to matched kidney tissue and urine samples from a control group (i.e. without chronic kidney disease). Notably however, our biopsy cohorts included participants with a wide range in age (19 years to 81 years old) and renal function (eGFR ranging from 8 to 133mls/min), hence the relatively wide range in proportions of p21+Ki67- senescent epithelia observed. We used a patient cohort recruited from secondary care nephrology services at risk of CKD progression (baseline eGFR < 60 mls/min or ACR > 30mg/mmol) and 3-year follow-up period, limiting the generalizability of these results to those with lower risk of progression. Larger studies that include patients spanning a complete range of ethnicities and incorporating those with lower risk of CKD progression and longer follow-up will therefore be required

				to define the utility of uCCR in these populations..
Interpretation	20	Give a cautious overall interpretation of results considering objectives, limitations, multiplicity of analyses, results from similar studies, and other relevant evidence	16	
Generalisability	21	Discuss the generalisability (external validity) of the study results	Pages 15 and 16.	We used a patient cohort recruited from secondary care nephrology services at risk of CKD progression (baseline eGFR < 60 mls/min or ACR > 30mg/mmol) and 3-year follow-up period, limiting the generalizability of these results to those with lower risk of progression.
**Other information**				
Funding	22	Give the source of funding and the role of the funders for the present study and, if applicable, for the original study on which the present article is based		All authors have completed ICMJE forms which included all sources of funding.

*Give information separately for cases and controls in case-control studies and, if applicable, for exposed and unexposed groups in cohort and cross-sectional studies.

Note: An Explanation and Elaboration article discusses each checklist item and gives methodological background and published examples of transparent reporting. The STROBE checklist is best used in conjunction with this article (freely available on the Web sites of PLoS Medicine at http://www.plosmedicine.org/, Annals of Internal Medicine at http://www.annals.org/, and Epidemiology at http://www.epidem.com/). Information on the STROBE Initiative is available at www.strobe-statement.org.
